# Efficient gene editing in induced pluripotent stem cells enabled by an inducible adenine base editor with tunable expression

**DOI:** 10.1038/s41598-023-42174-2

**Published:** 2023-12-11

**Authors:** Krittika Nandy, Dinesh Babu, Sonam Rani, Gaurav Joshi, Smitha Ijee, Anila George, Dhavapriya Palani, Chitra Premkumar, Praveena Rajesh, S. Vijayanand, Ernest David, Mohankumar Murugesan, Shaji R. Velayudhan

**Affiliations:** 1 Center for Stem Cell Research (A Unit of inStem, Bengaluru, India), Christian Medical College, Tamil Nadu, Vellore, 632002 India; 2 Department of Biotechnology, Thiruvalluvar University, Vellore, Tamil Nadu 632115 India; 3 Department of Haematology, Christian Medical College, Vellore, Tamil Nadu 632004 India; 4 Sree Chitra Tirunal Institute for Medical Sciences and Technology, Thiruvananthapuram, Kerala 695011 India

**Keywords:** Genetic engineering, Induced pluripotent stem cells

## Abstract

The preferred method for disease modeling using induced pluripotent stem cells (iPSCs) is to generate isogenic cell lines by correcting or introducing pathogenic mutations. Base editing enables the precise installation of point mutations at specific genomic locations without the need for deleterious double-strand breaks used in the CRISPR-Cas9 gene editing methods. We created a bulk population of iPSCs that homogeneously express ABE8e adenine base editor enzyme under a doxycycline-inducible expression system at the AAVS1 safe harbor locus. These cells enabled fast, efficient and inducible gene editing at targeted genomic regions, eliminating the need for single-cell cloning and screening to identify those with homozygous mutations. We could achieve multiplex genomic editing by creating homozygous mutations in very high efficiencies at four independent genomic loci simultaneously in AAVS1-iABE8e iPSCs, which is highly challenging with previously described methods. The inducible ABE8e expression system allows editing of the genes of interest within a specific time window, enabling temporal control of gene editing to study the cell or lineage-specific functions of genes and their molecular pathways. In summary, the inducible ABE8e system provides a fast, efficient and versatile gene-editing tool for disease modeling and functional genomic studies.

## Introduction

Human induced pluripotent stem cells (iPSCs) are a powerful platform for disease modeling and drug discovery. However, iPSC-based disease modeling is challenged by variability in pluripotency levels among iPSC lines, which can affect the phenotype of the differentiated cells^1^. This variability is caused by differences in the genetic backgrounds of donors and genetic and epigenetic alterations, even among iPSC lines derived from the same donor^2^. Gene-editing methods can be used to repair disease-associated mutations in patient-derived iPSCs or introduce them in normal iPSCs to generate isogenic mutant and wild-type iPSC lines^3–6^. Such isogenic cell line pairs with minimal genetic variations have been used for disease modeling and studying disease pathogenesis^3–6^. However, the current gene editing methods for generating isogenic iPSC lines are time-consuming and cumbersome. Rapid and efficient engineering of isogenic iPSC lines is necessary to accelerate in vitro disease modeling, research to understand disease pathogenesis and evaluation of the pathogenic risks associated with genetic variants identified through large-scale sequencing efforts^2^.

The most widely used CRISPR/Cas9 gene editing system comprises a chimeric guide RNA (gRNA) that provides target sequence specificity and a Cas9 protein that acts as a helicase and nuclease to unwind and cleave double-stranded DNA^7,8^. CRISPR-Cas9-based gene editing has been utilized to correct mutations in the patient-derived iPSCs^9–12^ or create mutations in normal iPSC lines^9,13,14^. However, correction or generation of specific point mutations by CRISPR-Cas9 mediated homology-directed repair (HDR)^10–12^ is ineffective, particularly in non-dividing cells. Moreover, the error-prone non-homologous end joining (NHEJ) repair mechanism outcompetes HDR at the targeted loci leading to undesired indel mutations at a substantial frequency that affects the potential benefit from corrected mutation^15,16^. Additionally, double-strand breaks (DSBs) by Cas9 nuclease can result in deleterious stochastic insertions, deletions, translocations and acquisition of potentially oncogenic mutations^17–23^. The ability to rapidly generate gene knockouts without DSBs will have significant implications in the iPSC-based research to elucidate the function of specific genes in development and disease.

Base editing, a recently introduced genome editing method, uses Cas9 nickase fused to a deaminase that generates a single-stranded nick and deaminates cytosine or adenine, resulting in transition mutations at the targeted regions. This gene editing strategy achieves more efficient and accurate single-base substitutions than HDR-mediated gene editing without deleterious DSBs^17–19^. The adenine base editor (ABE) almost exclusively generates A:T to G:C edits, while different generations of cytosine base editor (CBE) yield varying degrees of undesired products, in addition to the specific C:G to T:A edits that they are designed for^24^. There are more than 33,000 known point mutations linked to human diseases. ABE could theoretically be used to generate about 14% of these mutations and potentially correct nearly half of them^25^. Base editing has been utilized to generate isogenic mutant and wild-type iPSC pairs^24–30^. Although 26–92% base conversions have been reported in iPSCs^26–28^, low transfection and transduction efficiencies in iPSCs result in a prolonged culture of cells under stress conditions, such as low-density plating, drug selection and flow sorting. Most protocols for base editing in iPSCs involve plasmid-based delivery of base editor and gRNAs, followed by time-intensive FACS sorting or antibiotic selection to enrich the edited cells and single-cell sorting to obtain clones with desired heterozygous or homozygous mutations. Although electroporation of base editor (BE) mRNA and gRNAs in iPSCs showed faster editing of the targets with 25–85% efficiency, single-cell sorting and screening were still required to enrich the cells with homozygous mutations^29,30^.

Cell lines with constitutive or inducible expression of gene editing enzymes have been found to enhance gene editing efficiency. iPSCs with doxycycline (Dox)-inducible Cas9 cassette at the adeno-associated virus integration site 1 (AAVS1) of chromosome 19 demonstrate homogeneous Cas9 expression and high-efficiency gene editing upon Dox supplementation^31^. In this study, we describe the generation of iPSCs with a Dox-inducible expression of ABE8e, an ABE variant with high specificity and efficiency of adenine base conversion^32^ with reduced RNA editing^32^, from the AAVS1 safe harbor site. The bulk population of cells with homogeneous Dox-inducible expression of ABE8e was obtained within two weeks. After introducing gRNAs in the Dox-treated cells, they showed robust and tunable expression with efficient controlled adenine base conversion without constitutive or high-level expression of editing enzymes, which can activate p53 levels in the cells^33,34^. Dox-inducible ABE8e (iABE8e) from the AAVS1 site provides temporal regulation of gene knockout in iPSCs. This is crucial when investigating genes with pleiotropic functions during differentiation, self-renewal and survival of iPSCs, as failure to regulate gene expression may obscure gene function analysis at later stages or hinder iPSC propagation. This approach allows for specific molecular pathway analysis in iPSCs while avoiding lineage bias, cellular transformation, and compensatory effects from other genes. We efficiently generated iPSCs with homozygous mutations in *KLF1*, *CDAN1* and *FANCA* genes without single-cell sorting and successfully applied the *FANCA*-edited cells for studying Fanconi anemia (FA) cellular phenotypes. The AAVS1-iABE8e iPSC-gRNA system also allows biallelic multiplexed editing with a single delivery of targeting gRNAs, which is crucial for studying polygenetic diseases.

## Methods

### iPSC generation and maintenance

An iPSC line generated from the blood cells of a normal donor^35^ and AAVS1-iABE8e iPSCs were cultured in mTeSR plus medium (STEMCELL Technologies, Cat. No. 100-0276) and on Matrigel® hESC-Qualified Matrix (Corning, Cat. No. 354277) coated plates. The medium was changed every alternative day, and the cells were passaged at 1:4 when they reached 70–80% confluency (~ 4–5 days).

### Generation of pAAVS1-iABE8e-PuroR plasmid

The pAAVS1-PDi-CRISPRn plasmid (a gift from Bruce Conklin, Addgene plasmid # 73500)^36^ was digested with *Age*I and *Pac*I (New England BioLabs) to replace Cas9 with ABE8e. The ABE8e sequence was amplified from ABE8e plasmid (a gift from David Liu, Addgene plasmid # 138489)^32^ as two fragments for cloning by Gibson assembly. The first PCR fragment of ABE8e was amplified using a forward primer containing a homologous overhang to the destination plasmid and a reverse primer with a homologous overhang to the second fragment of ABE8e. The second fragment of ABE8e was amplified using a forward primer with a homologous overhang to the first fragment and a reverse primer containing a homologous overhang to the destination plasmid. The primer sequences used for generating the PCR-amplified products are shown in Supplemental Table S1.

Gibson assembly was set up using the NEBuilder® HiFi DNA Assembly Master Mix (New England BioLabs, Cat. No. E2621L) with 0.025 pmol of the digested backbone and 0.05 pmol of each amplified fragment, following the manufacturer’s protocol. XL1-Blue competent cells (Agilent Technologies) were transformed with 2 μl of the assembled product, and plasmids were extracted from the bacterial clones. The presence of the ABE8e sequence in the final pAAVS1-iABE8e-PuroR plasmid was confirmed by *EcoR*I (New England BioLabs) digestion and Sanger sequencing. The insert sequence of the plasmid was also analyzed by next-generation sequencing. In brief, plasmid DNA was sheared, and the library was prepared using the NEBNext Ultra DNA Library preparation kit (New England BioLabs in which the sheared DNA was subjected to the enzymatic steps for repairing the ends and tailing with a dA-tail followed by ligation of adapter sequences. These adapter-ligated fragments were then purified using solid-phase reversible immobilization beads) to remove any unbound adapter molecules or impurities. The purified fragments were amplified via limited-cycle PCR to generate the final sequencing libraries for paired-end sequencing on the Illumina HiSeq. The resulting .fasta files were analyzed using a custom script.

### Generation of AAVS1-Tet-On-ABE8e iPSC line

A normal iPSC line (1 × 10^6^ cells) was electroporated with 4 µg of pAAVS1-iABE8e-PuroR donor plasmid and 2 µg each of pZT-AAVS1-R1 and pZT-AAVS1-L1 plasmids (Addgene plasmid # 52638 and 52637, gifts from Mahendra Rao and Jizhong Zou)^37^ using the Neon Transfection System (Thermo Fisher Scientific) with a single pulse of 1300 V for 30 ms. After 48 h, the cells were treated with progressively increasing concentrations of puromycin (0.25 µg/ml for 2 days, 0.5 µg/ml for 5 days and 1 µg/ml for 7 days). The puromycin-selected cells were cultured and cryopreserved for future experiments.

### Junction PCRs for the AAVS1 site integration of the transgene cassette

To detect the integration of Tet-On-ABE8e at the AAVS1 site, junction PCRs were performed using the primers shown in Supplemental Table S1.

### Karyotyping

Karyotyping of AAVS1-iABE8e iPSCs was performed 24 passages after their generation using standard protocols. Briefly, the iPSC colonies in culture were exposed to 200 ng/ml colcemid for 30 min and harvested as single cell suspension with TrypLE™ Express Enzyme (Thermo Fisher Scientific, Cat. No. 12604021). The cell pellet was treated with 0.075 M KCl solution for 12 min at 37 °C and then fixed using modified Carnoy's fixative (methanol and acetic acid in a 3:1 ratio), followed by centrifugation at 1000 rpm for 10 min at room temperature. The fixed cells were resuspended in 5 ml of modified Carnoy's fixative, spread onto a slide, and then stained using standard cytogenetics protocols. Images were acquired using AxioImager A1 (Carl Zeiss, Germany) and analyzed using Ikaros Software (Metasystems, Germany). The size of the detected structural abnormality was > 3–10 Mb, and the band level was 400 bphs.

### Immunofluorescence

For the detection of the Dox-inducible expression of ABE8e in AAVS1-iABE8e iPSCs, the cells were seeded on Matrigel-coated glass bottom plates (Ibidi) with and without Dox supplementation. After 48 h, the cells were fixed with 4% paraformaldehyde at room temperature for 20 min. The fixed cells were blocked and permeabilized with a buffer containing 1% BSA, 5% FBS and 0.2% Triton X-100 for 45 min and incubated with anti-SpCas9 antibody (Cell Signaling Technology Cat. No. 14697) at a 1:200 dilution in the blocking buffer overnight at 4 °C, followed by incubation with goat anti-mouse IgG-Alexa Fluor™ 488 secondary antibody (Thermo Fisher Scientific Cat. No. A-11029) at a dilution 1:400 for 2 h at room temperature (RT). The nuclei were stained with 14.3 µM DAPI for 10 min at RT. Images were captured using an Olympus FV1000 confocal microscope (Olympus, Japan).

For pluripotency marker expression, AAVS1-iABE8e iPSCs were cultured on Matrigel-coated 6-well plates, and a similar procedure was followed. For surface markers, cells were not permeabilized. Imaging was done under a fluorescence microscope (DMI6000B, Leica Microsystems) with appropriate filters. The primary antibodies were from the StemLight™ Pluripotency Antibody Kit (Cell Signaling Technology, Cat. No. 9656). The secondary antibodies were anti-mouse or anti-rabbit IgG conjugated with Alexa Fluor 488 or Alexa Fluor 594 (Cat. No. A-11029, A-11032, A-11034 and A11037, Thermo Fisher Scientific).

### Trilineage differentiation

Trilineage differentiation was performed using the STEMdiff™ Trilineage Differentiation Kit (STEMCELL Technologies, Cat. No. 05230), as per the manufacturer’s protocol, and immunofluorescence was performed in the differentiated cells to detect the expression of PAX6 (ectoderm), SOX17 (endoderm) and Brachyury (mesoderm).

### Western blot

Whole-cell lysates were prepared using radioimmunoprecipitation assay (RIPA) buffer (150 mM sodium chloride, 1% Triton X-100, 0.5% sodium deoxycholate, 0.1% SDS, and 50 mM Tris, pH 8) containing Halt™ Protease and Phosphatase Inhibitor Cocktail (Thermo Fisher Scientific) and phenylmethanesulfonyl fluoride (Sigma Aldrich, Cat. No. 78830). The whole cell lysates (20 μg) were loaded on a 7% sodium dodecyl sulfate–polyacrylamide gel electrophoresis (SDS–PAGE) gel, and a western blot was carried out using mouse anti-Cas9 (Thermo Fisher Scientific), anti-FANCA (Cell Signaling Technology), mouse anti-FANCD2 (Santa Cruz Biotechnology) or anti-Actin (BD biosciences) primary antibodies and horseradish peroxidase-conjugated anti-mouse IgG (H + L) (Cell Signaling Technology) or anti-rabbit IgG (Thermo Fisher Scientific) secondary antibodies. The signal was detected using the Westar Supernova XLS3 Chemiluminescence detection kit (Cyanagen) and FluorChemE chemiluminescence detection system (Protein Simple). For the FANCD2 western blot, iPSCs were treated with 2 mM Hydroxyurea for 24 h before the cell lysates were prepared.

### Quantitative real-time PCR analysis

Total RNA was extracted from AAVS1-iABE8e iPSCs using NucleoZOL (Takara Bio Inc), and reverse transcription was carried out with 1 μg of total RNA using the PrimeScript™ RT-PCR Kit (Takara Bio Inc), following the manufacturer’s instructions. Quantitative RT-PCR was set up with SYBR Premix Ex Taq II (Takara Bio Inc) using the primers in Supplemental Table S1. Data were analyzed with QuantStudio 6 Flex real-time PCR systems (Thermo Fisher Scientific).

### Base editing in AAVS1-iABE8e iPSCs by electroporation of modified gRNAs

The gRNAs for each target were manually designed, and then BE-Hive^38^ was used to predict their overall adenine conversion efficiencies (average, below average and above average) in HEK293T cells. AAVS1-iABE8e iPSCs were treated with 0.5 µg/ml or 0.06 µg/ml Dox (Sigma-Aldrich) for 48 h before electroporation. 100 pmol of chemically modified gRNAs (Synthego) were electroporated into 1 × 10^6^ cells pre-treated with Revitacell Supplement (Gibco, Cat. No. A2644501) for 1 h. The treated cells were suspended in 100 µl electroporation Buffer R and electroporated using the Neon Transfection System (Thermo Fisher Scientific) at a single pulse of 1300 V for 30 ms. The cells were then plated with Revitacell Supplement (Thermo Fisher Scientific) without antibiotics for a day, following which the Dox treatment was resumed. After 3 to 5 days, PCRs were carried out using specific primers (Supplemental Table S1) to amplify the targeted genomic regions flanking the gRNA binding sites, followed by Sanger DNA sequencing of the amplicons. The percentages of base conversions were calculated using EditR^39^.

### Editing with lentiviral gRNAs

The gRNAs for ABE (Supplemental Table S1) were cloned into *BsmBI* digested pLKO5.sgRNA.EFS.GFP vector (a gift from Benjamin Ebert, Addgene plasmid # 57,822) as described earlier^31^. Plasmids containing the correct gRNA sequences were transfected into HEK293T cells along with the lentiviral envelope plasmid pMD2.G and the packaging plasmid psPAX2 (Addgene plasmid # 12259 and 12260, gifts from Didier Trono). The viral supernatants collected after 48 h and 72 h were pooled and stored as aliquots at − 80 °C^40^. iPSC colonies at 50% confluency in the wells of a 12-well plate were transduced with 200 µl of thawed virus mixed with 800 µl of iPSC culture medium, and the complete medium was changed after 6 h. The EGFP + transduced cells were flow-sorted after 6 days and treated with 0.5 µg/ml Dox for 3 days with editing quantification.

### Next generation sequencing for quantification of base editing

Editing was also confirmed by amplicon NGS. Approximately 200 bp surrounding the edited site was amplified using specific primers that contained sequences homologous to the secondary primers (Supplemental Table S1). The amplified products were then subjected to a secondary PCR using primers containing sample-specific barcodes. After adapter ligation, the fragments were sequenced for 2 × 150 paired-end reads with the Illumina HiSeq X sequencing system. Frequencies of editing outcomes were quantified using CRISPResso2^41^ and the A-to-G substitutions in the editing window (at positions 3–11 of the gRNA binding site, considering PAM at positions 21–23) were counted as base edits for total edit quantification.

### Off-target analysis

Off-target regions for *FANCA* L1082P gRNA were identified by CRISPOR^40^ and the genomic regions of the top 15 off-targets with the highest Cutting Frequency Determination (CFD) scores were PCR amplified and subjected to Sanger sequencing. The data were analyzed by EditR^39^. For exonic regions, the amino acid changes due to off-target conversions were analyzed by VarSome^42^ to predict their pathogenicity.

### Cell cycle analysis

The AAVS1-iABE8e iPSCs cultured in the presence and absence of Dox were electroporated with 100 pmol of *FANCA* gRNA. After 5 days, when the cells achieved 60% –70% confluency, they were analyzed using ClickIT EdU flow cytometry assay kit (Life Technologies). The cells were treated with 5 ng/ml Mitomycin C (MMC) for 16 h before EdU treatment. The iPSC colonies were treated with 100 µM EdU for 1 h and then dissociated into single-cell suspension by TrypLE Express (Thermo Fisher Scientific) and were fixed with 4% paraformaldehyde for 15 min. The fixed cells were permeabilized using a buffer containing saponin (provided in the kit) for 30 min. The fixed and permeabilized cells were treated with ClickIT reaction buffer containing Alexa Fluor™ 647 picolyl azide for 30 min at room temperature and then stained with DAPI for 2 h at 4 °C. The cells were resuspended in PBS, and cell cycle analysis was performed using FACSCelesta flow cytometer (BD Bioscience) and the FlowJo software (BD Bioscience) was used to analyze the data.

### Biosafety and ethics

All methods used in this study were carried out in accordance with the relevant guidelines and regulations approved by the Institutional Review Board (IRB), Christian Medical College, Vellore. Lentiviral production was performed with the biosafety protocols approved by the institutional biosafety committee (IBSC) of Christian Medical College, Vellore. The normal iPSC line used in this study was previously reported and it was generated after obtaining informed consent from the donor.

## Results

### Generation of AAVS1-iABE8e iPSCs

**Figure 1:**
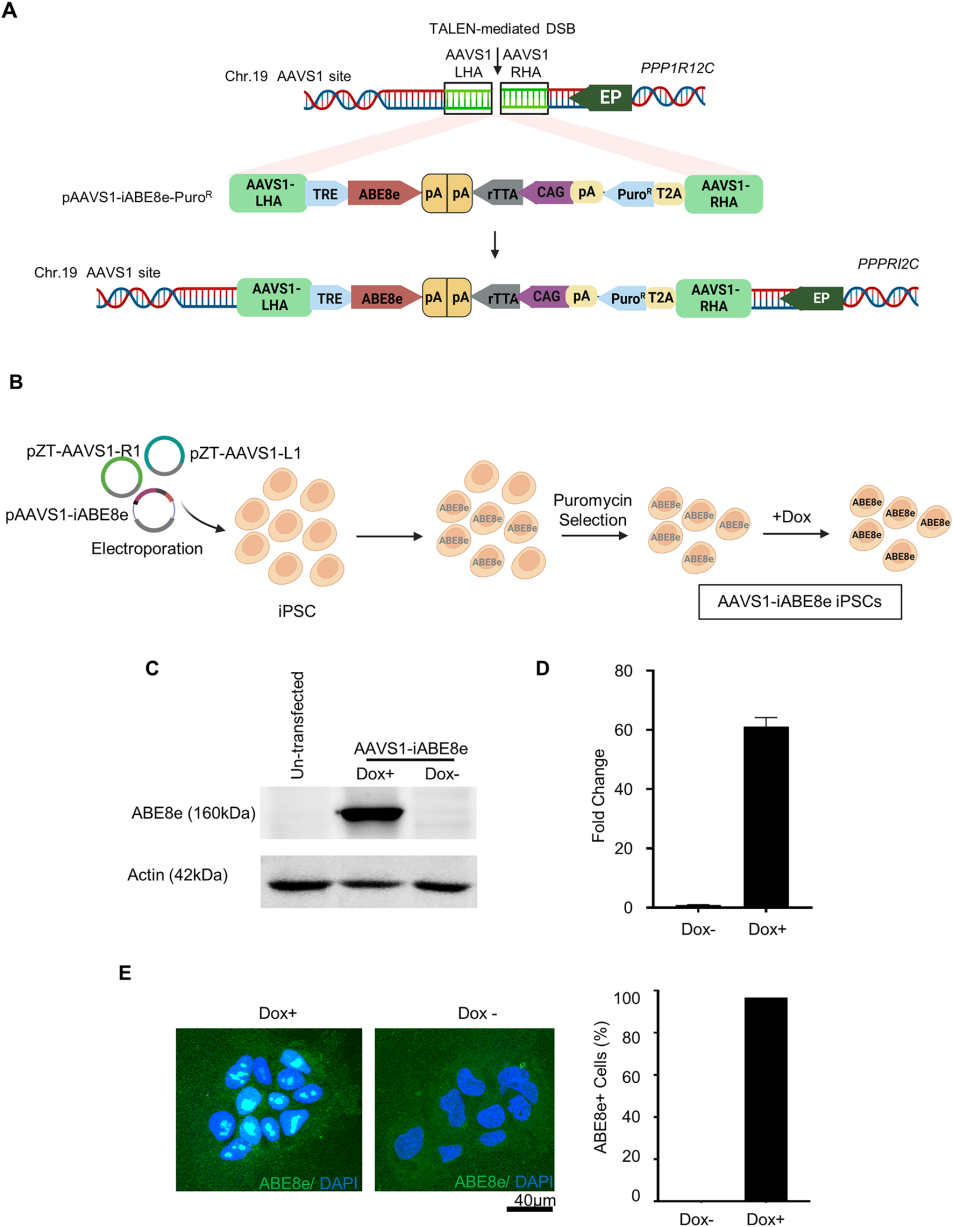
Generation and characterization of AAVS1-iABE8e iPSCs. **(A)** Schematic overview depicting the integration of a doxycycline (Dox)/tetracycline-inducible (Tet-on) ABE8e (iABE8e) cassette at the AAVS1 safe-harbor site on chromosome 19. The integration was facilitated by creating a double-strand break (DSB) using TALEN arrays, followed by HDR with the donor plasmid, pAAVS1-iABE8e-PuroR, which contains Tet-On-ABE8e cassette and puromycin resistance gene (PuroR). LHA and RHA represent the left and right homology arms in the donor plasmid that facilitate integration at the AAVS1 site. The expression of PuroR is driven by the endogenous *PPP1R12C* promoter (EP), while the expression of the reverse tetracycline-controlled transcriptional activator (rtTA) is driven by the constitutively expressed CAG promoter. The inducible expression of ABE8e is controlled by the tetracycline response element (TRE), which is activated by the addition of Dox in the culture medium. **(B)** To generate AAVS1-iABE8e iPSCs, plasmids containing TALEN arrays (pZT-AAVS1-R1 and pZT-AAVS1-L1) were co-electroporated with the donor plasmid (pAAVS1-iABE8e-PuroR) into a normal iPSC line followed by selection with puromycin to select for cells with correct transgene integration. **(C)** Western blot analysis showing ABE8e protein expression levels before and after treatment with 0.5 µg/ml Dox for 48 h. (**D**) qPCR analysis showing ABE8e expression levels before and after treatment with 0.5 µg/ml Dox for 48 h. The data represent the mean ± SD of three independent experiments. (**E**) Immunostaining to detect ABE8e expression (Green) in AAVS1-iABE8e iPSCs with (Dox +) and without (Dox-) Dox treatment. The percentage of cells expressing ABE8e was quantified across 15 imaged fields and 298 individual cells for the Dox-treated (Dox +) samples and 5 imaged fields and 88 individual cells for the control untreated (Dox-) samples. Nuclei were stained with DAPI (blue). Scale bars: 40 µm. (The western blot images are cropped and the original western blots are presented in Supplemental Figure S8B).

ABE8e adenine base editor variant has been reported to exhibit superior adenine base conversion efficiency and reduced RNA editing compared to other variants^32^. We utilized a previously reported TALEN-based approach, known for the efficient generation of reporter cell lines^43^, to generate iPSCs with a Dox-inducible ABE8e expression cassette integrated at the AAVS1 safe harbor genomic site on human chromosome 19. A donor plasmid containing the puromycin resistance gene and a Dox-inducible ABE8e (iABE8e) expression cassette, flanked by homologous arms for HDR at the target site, was generated (Fig. 1A). The expression of the integrated puromycin-resistance gene and ABE8e at the AAVS1 site was driven by endogenous PPP1RI2C promoter and tetracycline-inducible promoter, respectively (Fig. 1A). To induce DSBs and enable integration at the AAVS1 site, we electroporated a well-characterized normal iPSC line that was previously generated in our laboratory with TALEN expression plasmids and the donor plasmid (Fig. 1A). The nucleofected cells were cultured in the presence of puromycin to select the cells with stable integration of the iABE8e cassette and puromycin resistance gene near the PPPRI2C promoter (Fig. 1B). We could obtain 18–24 puromycin-resistant colonies from 1 million nucleofected iPSCs. The Dox-inducible expression system, based on the constitutively expressed transcriptional activator (rtTA) binding to tetracycline-response element (TRE) promoter in the presence of tetracycline or its orthologue Dox, allows for the controlled and reversible expression of transgenes. The modified promoter (TRE-tight) and the mutated reverse transcriptional activator (rtTA-M2) favored the tight dose-dependent expression of transgenes^44,45^.

The transgene integration at the AAVS1 site in the puromycin-selected cells was confirmed by junction PCRs (Supplemental Figs. S1A and S8A). The resultant AAVS1-iABE8e-iPSCs expressed high levels of ABE8e only in the presence of Dox as measured by immunoblot analysis with a wild-type anti-Cas9 antibody (Fig. 1C and Supplemental Fig. S8B) and qPCR (Fig. 1D). Immunofluorescence analysis revealed that > 95% of the puromycin-selected cells expressed ABE8e (Fig. 1E), which facilitated the use of these cells for ABE experiments without single-cell sorting to generate iPSC clonal lines expressing ABE8e. Further, ABE experiments demonstrated that these cells could generate mutations with ~ 100% efficiency for some targets, confirming the homogeneous expression of ABE8e in the puromycin-resistant cells (Fig. 3C). These cells had normal karyotypes, retained the expression of pluripotency markers and the ability for trilineage differentiation (Supplemental Fig. SIB–D). A bulk population of AAVS1-iABE8e iPSCs with uniform inducible expression of ABE8e could be generated within two weeks without requiring single-cell sorting and cloning to generate the cell line.

### Temporal and tunable ABE8e expression in AAVS1-iABE8e iPSCs

Controlled expression of gene editing enzymes is crucial for selectively targeting genes in specific cell types or lineages derived from iPSCs. The ability to regulate the expression of gene editing enzymes avoids unwanted cellular responses and achieves precise gene editing outcomes. Further, constitutive and high levels of expression of gene editing enzymes can activate p53 and lead to adverse consequences in the cells^33,34^. Therefore, we performed experiments to determine the optimal Dox concentration and treatment duration required to achieve maximum ABE8e expression for obtaining efficient adenine base conversion.

**Figure 2:**
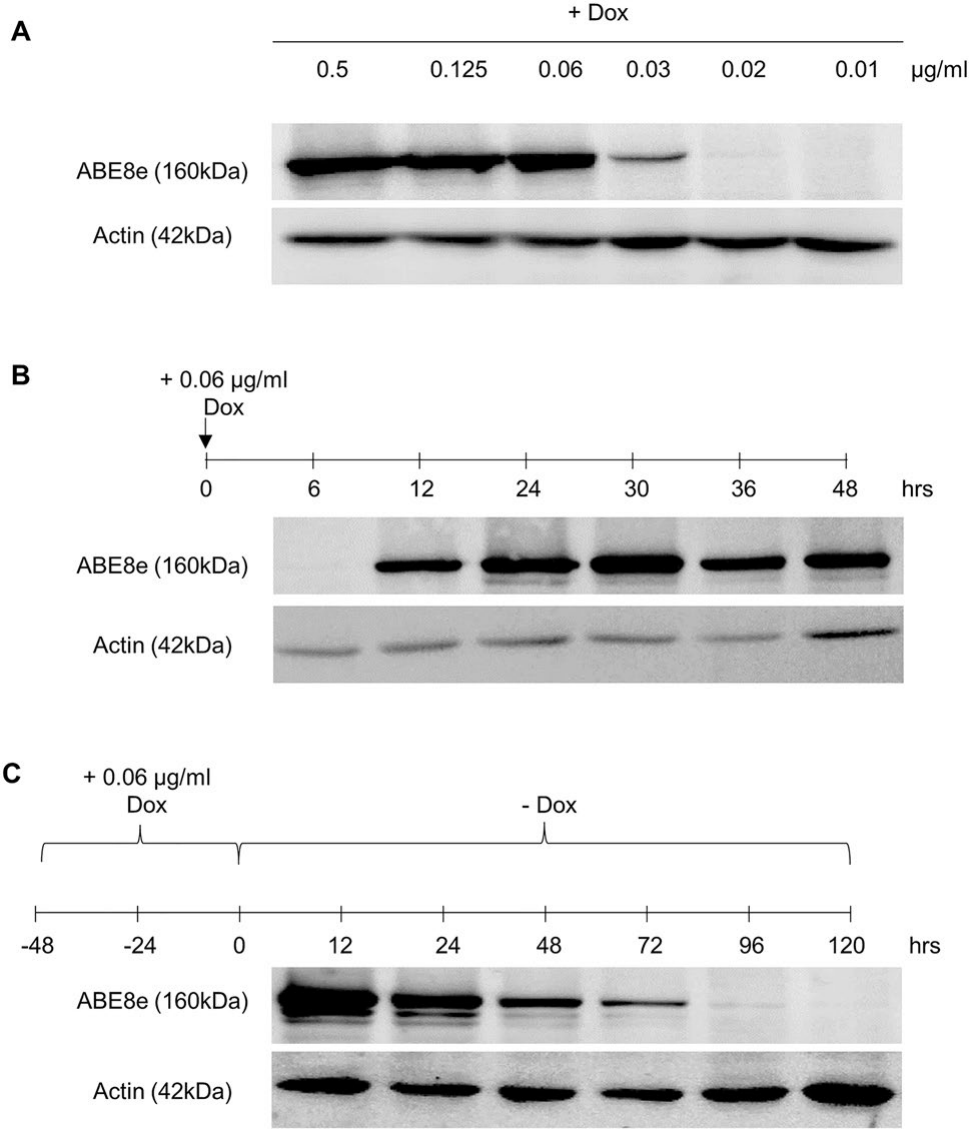
Analysis of Dox-inducible ABE8e expression in AAVS1-iABE8e iPSCs. **(A)** Western blot analysis of ABE8e expression in AAVS1-iABE8e iPSCs after treatment with various concentrations of Dox (0.5, 0.125, 0.06, 0.03, 0.02, and 0.01 µg/ml) for 48 h to determine the minimum concentration required for stable ABE8e expression. ABE8e expression was detected with an anti-SpCas9 antibody, and an anti-Actin immunoblot was used as a control. **(B)** Time course analysis of the stability of ABE8e expression in AAVS1-iABE8e iPSCs treated with 0.06 µg/ml Dox. ABE8e expression was quantified by western blotting 6, 12, 24, 30, 36, and 48 h after Dox treatment. **(C)** Temporal analysis of ABE8e expression in AAVS1-iABE8e iPSCs treated with a single dose of 0.06 µg/ml Dox for 48 h, followed by withdrawal of Dox. (The western blot images are cropped and the original western blots are presented in Supplemental Figure S9A–C).

When the AAVS1-iABE8e iPSCs were cultured in the presence of Dox concentrations ranging from 0.01 to 0.5 µg/ml for 48 h, we found that ABE8e expression could be detected at Dox concentrations as low as 0.03 µg/ml, and stable ABE8e expression could be achieved with a Dox concentration of 0.06 µg/ml. ABE8e expression levels remained constant even at higher Dox concentrations (Fig. 2A and Supplemental Fig. S9A). We also observed that ABE8e expression could be detected within 12 h of treating the cells with 0.06 µg/ml Dox, and the expression stabilized at 24 h and remained unchanged for 48 h without further Dox supplementation (Fig. 2B and Supplemental Fig. S9B). When Dox was withdrawn after 48 h of supplementation, ABE8e expression decreased to undetectable levels after 4 days (Fig. 2C and Supplemental Fig. S9C). In summary, the ABE8e transgene integrated at the AAVS1 site exhibited tight and tunable expression suitable for temporal adenine base editing applications in iPSCs.

### High editing efficiency of inducible ABE8e at single and multiple targets

To assess the effectiveness of AAVS1-iABE8e iPSCs in generating mutations in target genes, we designed gRNAs to introduce four mutations associated with human diseases causing severe anemia: *KLF1* Y290H and L300P and *CDAN1* T884A and F360L. The gRNAs were manually designed to position the targeted mutations within the editing windows, as shown in Supplemental Fig. S2A–D. We employed BE-Hive^38^, an in silico tool to predict the overall efficiency of the gRNAs and the conversion percentages of the target and bystander adenines in the editing windows. To avoid undesirable mutations by bystander effects^25^, we preferred the gRNAs without additional adenines in the editing window or those with bystander conversions resulting in silent (synonymous) missense mutations. In cases where bystander editing was unavoidable, we chose a gRNA that converted the target base at a higher rate than the bystander bases. The editing window of the gRNA designed for *KLF1* Y290H had only one targeted adenine, thus eliminating any bystander conversions. The gRNA for *KLF1* L300P and *CDAN1* T884A contained an additional adenine base in the editing window, which resulted in a silent mutation without any amino acid change. For *CDAN1* F360L, only a low conversion efficiency gRNA could be obtained, and the editing window of this gRNA had two additional adenine bases, which could result in multiple combinations of amino acid changes (Supplemental Fig. S2).

We first determined the optimal Dox concentration required for efficient base conversion using a gRNA with a single adenine in the editing window to generate *KLF1* L296P mutation (Supplemental Fig. S11). The AAVS1-iABE8e iPSCs were treated with various concentrations of Dox: 0.03 µg/ml, 0.1 µg/ml, 0.5 µg/ml, and 1 µg/ml. Although a detectable level of ABE8e expression was observed with 0.03 µg/ml Dox (Fig. 2A), no editing was observed at the *KLF1* L296P target with this concentration (Supplemental Fig. S11) and we obtained a very low editing percentage (4% to 6%) with 0.1 µg/ml Dox. Significant editing was observed only after treatment with 0.5 µg/ml Dox (Supplemental Fig. S11), suggesting that more than a specific expression level of the ABE8e is essential for the formation of ribonucleoprotein (RNP) complexes with the nucleofected gRNAs to induce base editing.

**Figure 3:**
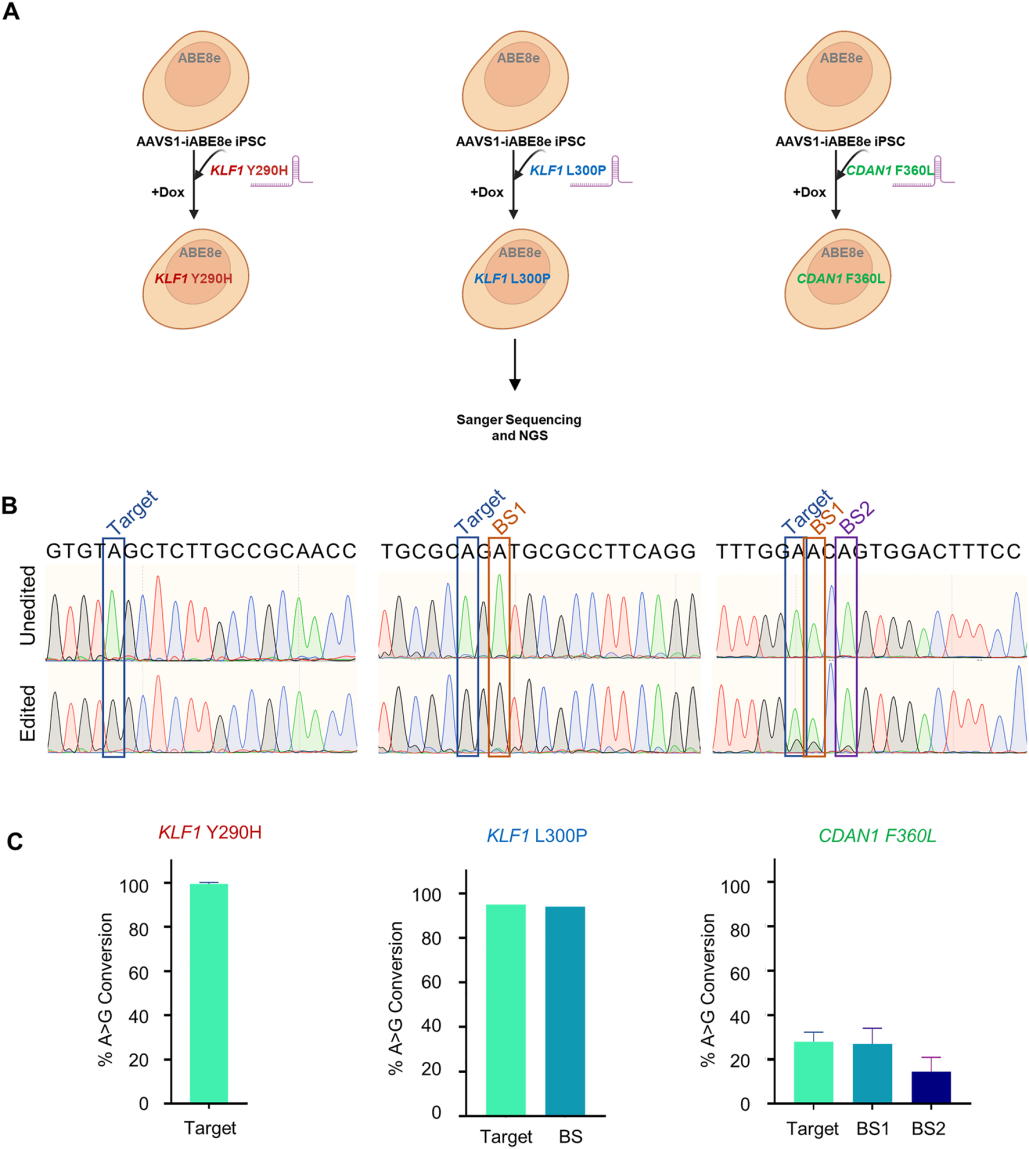
Single locus adenine base editing in AAVS1-iABE8e iPSCs. **(A)** Schematic of adenine base editing in AAVS1-iABE8e iPSCs by electroporation of gRNAs separately. Synthetic gRNAs for *KLF1* Y290H, *KLF1* L300P and *CDAN1* F360L were electroporated into AAVS1-iABE8e iPSCs separately after treating the cells with Dox to activate ABE8e expression. The base editing was quantified by NGS and Sanger sequencing. (**B**) Sanger sequencing results showing A to G conversions in the editing windows of the gRNAs. (**C**) Bar graphs showing the percentages of A to G conversions quantified by Sanger sequencing (analyzed by EditR). The data represent the mean ± SD of two independent experiments. BS1 and BS2 denote bystander mutations.

After culturing AAVS1-iABE8e iPSCs in the presence of Dox for 48 h to attain stable ABE8e expression, the cells were electroporated with modified synthetic gRNAs individually to generate *KLF1* Y290H, *KLF1* L300P and *CDAN1* F360L mutations. Base conversion analysis was performed with the DNA extracted 5 days after electroporation. Transfection with the gRNA designed for generating *KLF1* Y290H resulted in 100% editing at the target base without bystander effect as expected, while the gRNA for *KLF1* L300P converted the target base with 100% efficiency and the bystander base with 94.1% efficiency. The *KLF1* Y290H mutation persisted in the mutant iPSCs with ~ 95% editing over 30 days (Supplemental Figure S14), suggesting the permanent installation of mutations in iPSCs by base editing. The low-efficiency gRNA for *CDAN1* F360L converted 29% of the target base and 26% and 15.5% of the two bystander bases (Fig. 3A–C and Supplemental Fig. SS3A–C). Similar results were obtained when iPSCs were transfected with ABE8e mRNA (Supplemental Fig. S12) validating that AAVS1-iABE8e iPSC line performs as efficiently as already reported protocols, without the added cost of mRNA production.

**Figure 4:**
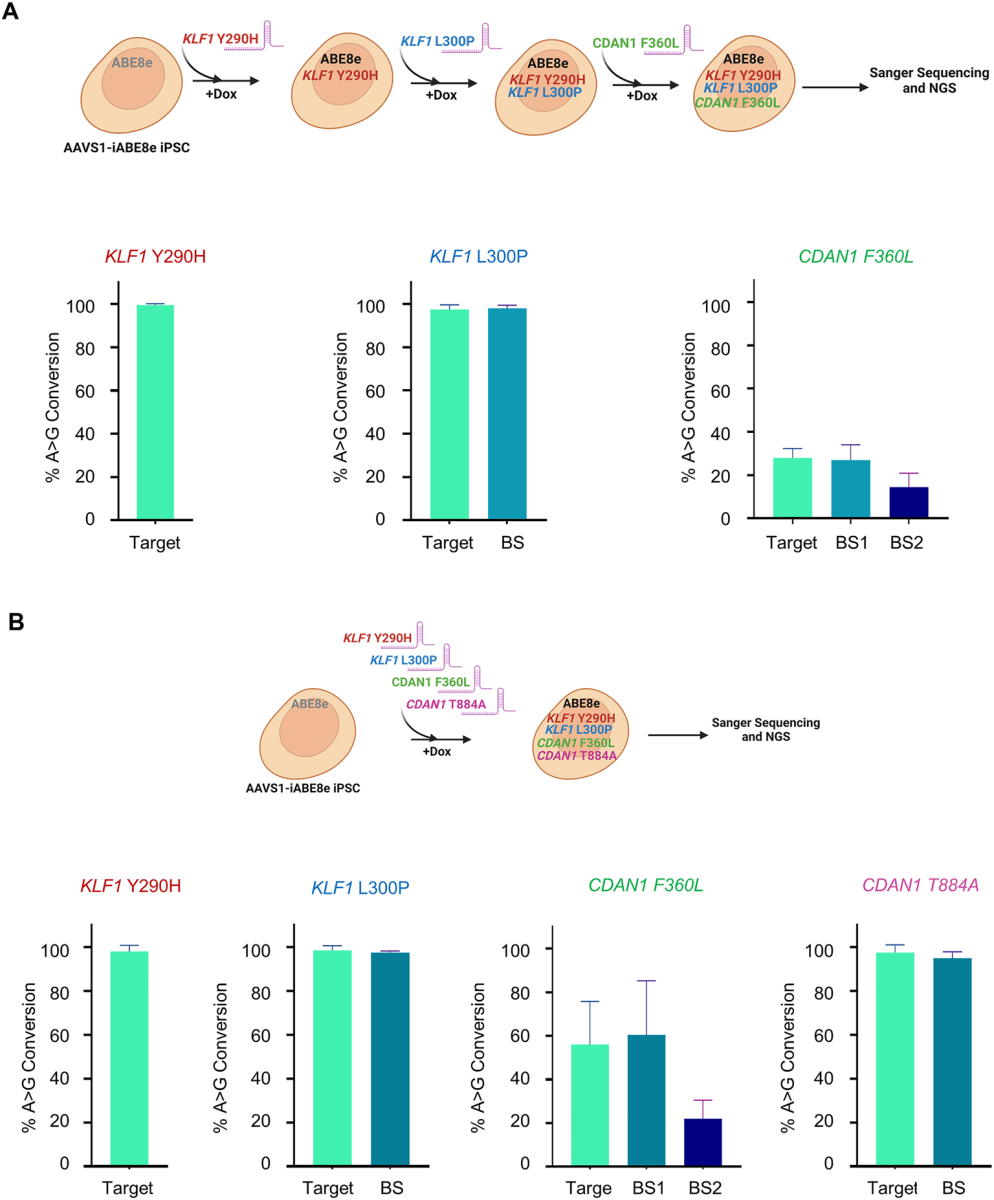
Adenine base editing of multiple genes in AAVS1-iABE8e iPSCs. **(A)** (Upper Panel) Multiplex temporal editing of genes in AAVS1-iABE8e iPSCs by sequential electroporation of gRNAs. Synthetic gRNAs to create *KLF1* Y290H, *KLF1* L300P and *CDAN1* F360L mutations were electroporated sequentially into AAVS1-iABE8e iPSCs treated with Dox to induce ABE8e expression. Base editing was quantified by NGS and Sanger sequencing. (Lower Panel) The A to G transitions at the targeted loci quantified by Sanger sequencing (analyzed by EditR). The data are presented as the mean ± SD of two independent experiments **(B)** (Upper Panel) Multiplex simultaneous editing of genes in AAVS1-iABE8e-iPSCs by co-electroporation of multiple gRNAs. Synthetic gRNAs to create *KLF1* Y290H, *KLF1* L300P, *CDAN1* F360L and *CDAN1* T884A mutations were electroporated together into AAVS1-iABE8e iPSCs treated with Dox. (Lower Panel) The bar graphs show A to G conversions quantified by Sanger sequencing (analyzed by EditR). The data represent the mean ± SD of two independent experiments. BS, BS1 and BS2 denote bystander mutations.

To study polygenic diseases caused by mutations in multiple genes and the effect of multiple genetic variations on the phenotype of monogenic disorders, it is crucial to generate iPSCs with multiple mutations. We electroporated AAVS1-iABE8e iPSCs with three gRNAs sequentially to generate *KLF1* Y290H, *KLF1* L300P and *CDAN1* F360L mutations (Fig. 4A and Supplemental Fig. S4). Subsequently, we electroporated the cells with 4 gRNAs simultaneously for multiplex editing to create *KLF1* Y290H, *KLF1* L300P, *CDAN1* T884A and *CDAN1* F360L mutations (Fig. 4B and Supplemental Fig. S5). The base conversion percentages obtained at the four targets in multiple editing were similar to those obtained by individual electroporation (Fig. 3A–C). Altogether, our data showed that efficient single and multiple genomic base editing conversions could be achieved using AAVS1-iABE8e iPSCs.

### Editing in AAVS1-iABE8e iPSC with lentiviral delivery of gRNAs

Lentiviral plasmids with a large number of gRNAs are used for pooled library gene editing screenings for simultaneous editing of several genes for the identification of genes and genetic elements essential for cellular processes or phenotypes^46^. To evaluate the feasibility of lentiviral gRNA vectors for base editing in AAVS1-iABE8e iPSCs, the cells were transduced using gRNAs with varying predicted efficiencies to target three genomic sites in the *HBG1* and *HBG2* promoter sequences (Supplemental Fig. S6A). The gRNA designed for site 1 was predicted to convert two adenines (A_3_ and A_9_) with editing efficiencies of 56.6% and 18.8%, respectively, while the one for site 2 was predicted to convert 2 adenines (A_4_ and A_5_) with efficiencies of 41.3% and 29.6% efficiencies, respectively. The gRNA designed for site 3 was predicted to convert 4 adenines (A_5_, A_8_, A_9_ and A_11_) with editing efficiencies of 7.4% (A_5_), 10.8% (A_8_), 18.1% (A_9_) and 27.8% (A_11_), respectively (Supplemental Fig. S6A). These gRNAs were cloned into the pLKO5.sgRNA.EFS.GFP lentiviral gRNA expression vector^47^, which also expresses enhanced green fluorescent protein (EGFP) reporter gene by EF1α core promoter.

**Figure 5:**
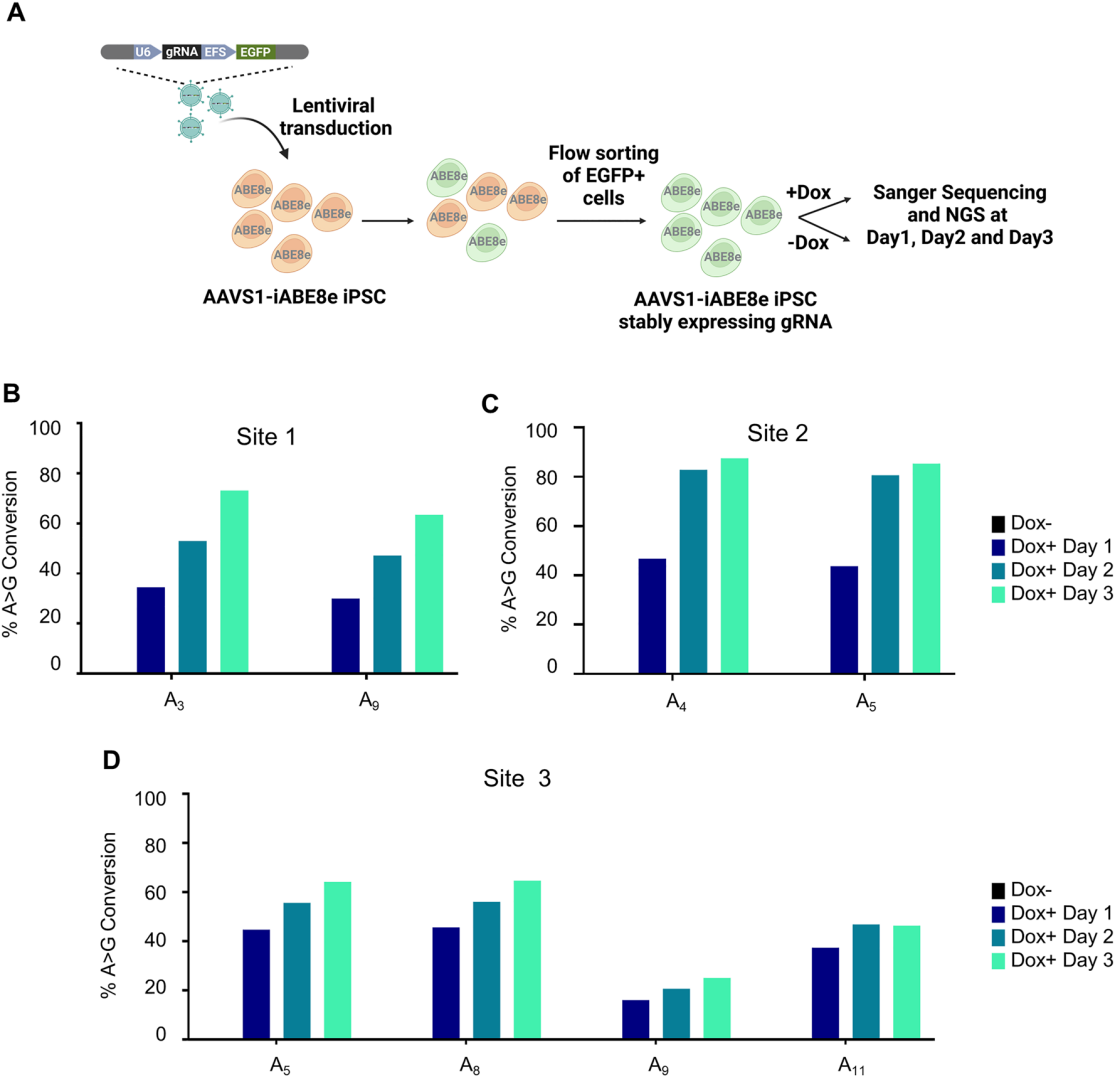
Adenine base editing in AAVS1-iABE8e iPSCs using lentiviral gRNAs. **(A)** Schematic representation of lentiviral gRNA-based adenine base editing. Three gRNAs targeting the *HBG1* and *HBG2* promoters were cloned into a lentiviral plasmid that also expresses EGFP. AAVS1-iABE8e iPSCs were transduced with the lentiviruses and the EGFP + cells were flow-sorted to enrich the transduced cells with integrated gRNAs. These cells were then treated with Dox, and quantification of editing in the *HBG1* and *HBG2* promoter regions was performed by NGS after 1, 2 and 3 days of Dox treatment. The Dox untreated cells (Dox-) were analyzed on day 3 of the respective dox-treated cells (Dox +) cells (**B)** gRNA against Site 1 converted 4 adenines (A_5_, A_8_, A_9_ and A_11_) **(C)** gRNAs against Site 2 converted two adenines (A_3_ and A_9_) and **(D)** gRNA against Site 3 converted 2 adenines (A_4_ and A_5_).

After 48 h of transduction, EGFP + cells were flow-sorted to obtain a population of cells with the integrated gRNAs, treated with 0.6 µg/ml Dox, and the editing was quantified by NGS over 3 days (Fig. 5A). The results showed that the percentages of base conversion in the experimental samples increased over time and reached efficiencies higher than those predicted by BE-Hive. The gRNA for site 1 edited the 2 adenines with 73.2% (A_3_) and 63.5% (A_9_) conversion and the gRNA for site 2 converted 2 adenines with 87.5% (A_4_) and 85.4% (A_5_) editing efficiencies by day 3. The editing window of the gRNA for site 3 had 4 adenines, which yielded 64.2% (A_5_), 64.7% (A_8_), 25.1% (A_9_) and 46.4% (A_11_) base conversions (Fig. 5B–D and Supplemental Fig. S6B). Overall, the experimental base conversion efficiency was significantly higher than the predicted rates. These results showed that the AAVS1-iABE8e iPSC line could be efficiently edited with lentivirally transduced stably expressing gRNAs, regardless of their predicted efficiencies.

### Recapitulation of Fanconi anemia (FA) cellular phenotypes in *FANCA* base edited AAVS1-iABE8e iPSCs

**Figure 6:**
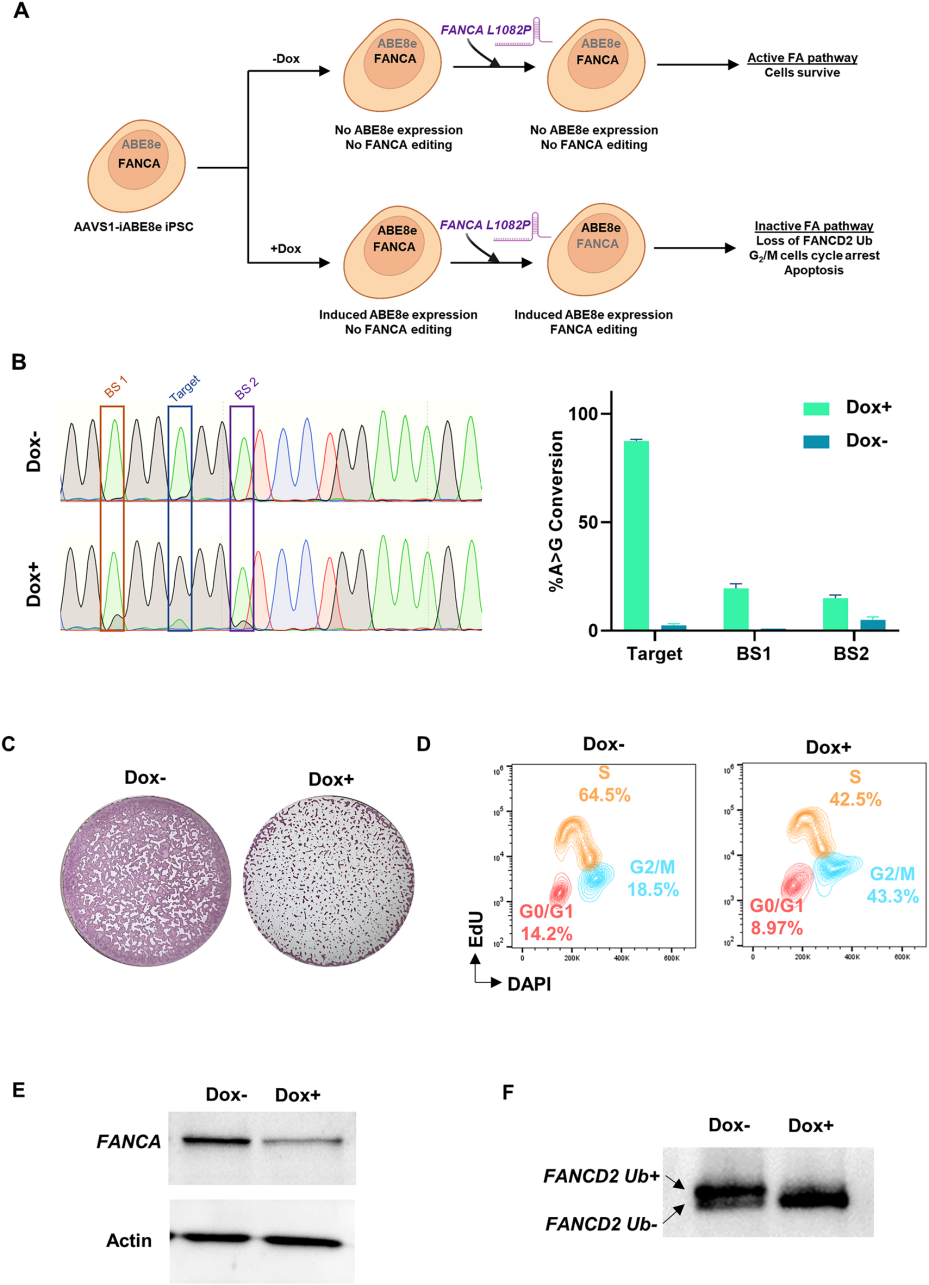
Recapitulation of Fanconi anemia (FA) cellular phenotypes in *FANCA* base edited in AAVS1-iABE8e iPSCs. **(A)** Schematic representation of *FANCA* editing in AAVS1-iABE8e iPSCs through electroporation of gRNA in the absence and presence of Dox that induces ABE8e expression and gene editing. **(B)** Quantification of base editing by Sanger sequencing and EditR analysis 3 days after electroporation of gRNA into AAVS1-iABE8e iPSCs. **(C-E)** Exhibition of FA cellular phenotypes in the AAVS1-iABE8e iPSCs after induction of *FANCA* editing: Progressive cell death estimated by alkaline phosphatase staining **(C)**, G_2_/M cell cycle phase arrest measured by cell cycle analysis **(D)** decreased expression of FANCA after Dox induced ABE8e editing **(E)** and lack of FANCD2 ubiquitination detected by western blot analysis **(F)**. (The western blot images are cropped and the original western blots are presented in Supplemental Figure S10A and B). BS1 and BS2 denote the bystander mutations.

Fanconi anemia (FA), which affects the hematopoietic system and causes bone marrow failure, is caused by mutations in the genes involved in the FA inter-strand cross-link (ICL) repair pathway^48^. The functional FA pathway is crucial for somatic cell reprogramming, making it challenging to generate patient-specific iPSCs without complementing the patient cells with the normal cDNA of the FA pathway genes that are defective in the cells^49^. To overcome this challenge of generating isogenic wild-type and mutant iPSCs for recapitulating FA cellular phenotypes, we attempted base-editing to create mutations in the *FANCA* gene in AAVS1-iABE8e iPSCs. It has been reported earlier that iPSCs with *FANCA* homozygous mutations (*FANCA* -/-) exhibit severe cell death, G_2_/M cell cycle phase arrest and loss of FANCD2 ubiquitination^50–52^. We performed ABE in the Dox-treated and untreated AAVS1-iABE8e iPSCs to create the *FANCA* L1082P mutation, which has been previously reported in some FA patients^53^ (Fig. 6A). Subsequently, we examined the *FANCA* homozygous mutant iPSCs for the previously described cellular phenotypes^50–52^.

We selected a gRNA predicted to have “above average” overall predicted efficiency of base conversion with 64.3%, 3.6% and 10.3% predicted base conversions of the target adenine, bystander 1 and bystander 2 adenines, respectively (Supplemental Fig. S7A). After supplementing with Dox for 5 days with electroporation during the Dox treatment, we achieved 87.5% conversion of the target adenine base to create L1082P mutation along with 19.5% and 15% of bystander conversions resulting in additional I1081T and L1083P mutations (Fig. 6B and Supplemental Fig. S7B and C). In contrast, cells that were not treated with Dox showed negligible conversions (Fig. 6B and Supplemental Fig. S7B and C). The bystander mutations, I1081T and L1083P, had Evolutionary Model of Variant Effect (EVE) scores^54^ of 0.563 and 0.795, respectively, suggesting a potential variant of unknown significance and a pathogenic variant, respectively. This combination of missense mutations resulted in a null-like *FANCA* genotype, leading to the loss of a functional FA pathway and subsequently causing significant cell death, as determined by alkaline phosphatase staining of Dox-treated cells (Fig. 6C). Over time, we observed a decrease in the percentage of mutant cells within the edited population (Supplemental Fig. S7D). Cell cycle analysis of the Dox-treated cells revealed a significant number of cells arrested at the G_2_/M phase in the electroporated iPSC population compared to the untreated cells (Fig. 6D). Furthermore, the expression of FANCA was significantly reduced in Dox-treated cells compared to untreated cells (Fig. 6E and Supplemental Fig. S10A) and FANCD2 monoubiquitination, a key event in the FA repair pathway, was also absent in the edited population as observed by the western blot analysis (Fig. 6F and Supplemental Fig. S10B).

Although the selected *FANCA* gRNA also created certain off-target base changes, 10 out of 15 off-targets with the highest Cutting Frequency Determination (CFD) scores were in the intergenic or intronic regions. The remaining 5 were in the protein-coding regions; 2 in the 3’ UTR and 3 in the exonic sequences (Supplemental Table S2). Experimental validation of the off-target conversions showed that the base conversions in the off-target sites ranged from 0 to 77% (Supplemental Fig. S13 and Supplemental Table S2). The conversions in the exonic regions ranged from 1 to 9%. Due to these low conversion percentages and the low to nil expression of the target proteins in iPSCs, the off-target editing was not expected to cause the cellular phenotype that we observed in the electroporated cells. We could conclude from these experiments that the mutant iPSC pool, mainly consisting of homozygous *FANCA* mutant cells, generated by base editing recapitulated important FA cellular phenotypes, indicating that this method is suitable for developing iPSC-based FA disease models.

## Discussion

More than 50% of the human disease-causing variants reported in ClinVar are single nucleotide mutations^25^. Thus, the ability to rapidly and accurately introduce or correct point mutations in iPSCs has significant implications for creating isogenic cell lines to study the function of specific genes in development and disease. Base editing is a novel gene editing tool that allows precise and efficient installation of point mutations without using commonly used low-efficiency HDR process to introduce single base pair changes^17,23^ that may cause deleterious by-products of Cas9-induced DSB-mediated genome editing^15,16,34,55^. Base-editing toolkits hold promise for gene therapy, including correcting disease-associated mutations and developing isogenic cell lines by introducing stop codons to generate isogenic gene knock-out iPSC lines^56^.

In this study, we report the rapid generation of iPSCs with the Dox-inducible expression of ABE8e, an ABE variant with high specificity and efficiency of adenine base conversion^32^ with reduced RNA editing^32^, from the AAVS1 safe harbor site. Gene editing using the AAVS1-iABE8e-gRNA system is easier to perform, faster and more cost-effective, and more efficient than other gene editing methods used in iPSCs. It also allows for multiplexed biallelic editing with a single delivery of targeting gRNAs, without the need for sequential retargeting, which is relevant for polygenic diseases^57^. We could successfully recapitulate the previously described FA cellular phenotypes in *FANCA* gene-edited iPSCs. FA disease is characterized by reduced hematopoietic cells due to bone marrow failure. FA disease modeling can be effectively accomplished through the utilization of iABE8e iPSCs. This will involve the initial step of lentiviral transduction with FANCA gRNAs in iABE8e iPSCs and subsequent temporal expression of ABE8e in the hematopoietic progenitors derived from the iABE8e cells. The high efficiency of this base editing method generates bulk-edited cells without requiring single-cell sorting and screening of multiple clones to identify those with homozygous mutations. Transgenes integrated at a genomic safe harbor locus, such as adeno-associated virus integration site 1(AAVS1) within the PP1R12C gene^58–63^, retain their transcriptional activity without significant silencing in almost all types of cells tested^61^. The high base editing efficiency we obtained may be due to the homogeneous expression of ABE8e from the AAVS1 site.

The development of base editing technology has significantly expanded the capability for rapid isogenic iPSC generation for disease modeling, drug screening and therapeutic applications^26–29,29,30,64,65^. However, most previous reports achieving significant base conversion in iPSCs have used plasmids to express gRNAs, flow sorting to enrich the transfected cells, and single-cell sorting to select the iPSC clones with homozygous and heterozygous mutations^27^. In a recent study, highly efficient (> 90%) cytosine base conversion at multiple loci has been reported to generate isogenic iPSC clones using a transient reporter for bulk enrichment of base-edited cell populations^56^. However, the efficiency of adenine base editing for multiple genes was unknown. AAVS1-iABE8e iPSCs that we generated showed robust and tunable ABE8e expression and efficient adenine base conversion within 3–5 days after nucleofection with gRNAs and Dox treatment. We could efficiently generate a pool of iPSCs that predominantly possessed homozygous mutations in three disease associated genes, *KLF1*, *CDAN1* and *FANCA*. The *FANCA* homozygous mutant iPSCs created through base editing successfully recapitulated salient cellular FA phenotypes that had been previously described in *FANCA*-/- iPSCs. Unlike homozygous missense mutations in *KLF1* and *CDAN1,* which had no impact on the survival of iPSCs, *FANCA* mutations resulted in significant cell death due to its crucial role as an essential gene for iPSCs. Furthermore, we also showed that lentiviral transduction of gRNAs in these cells could achieve a significant base conversion at the targeted genomic regions, which increased with increasing Dox exposure. Therefore, this cell line is suitable for pooled gRNA library screenings for elucidating the mechanisms governing cellular processes, such as lineage differentiation.

Although the ABE8e variant exhibits higher editing efficiency and low off-target activity compared to other ABE variants^32^, it still poses two major limitations, namely inducing A to G conversions at other adenines in the editing window, leading to bystander mutations and requiring NGG PAMs near the targeted regions, which restricts the number of mutations that could be generated. Bystander editing occurred in a large proportion of our bulk-edited cells, but we found that gRNAs could be chosen such that the bystander edits did not alter the amino acids and protein function. If the occurrence of bystander mutations that lead to amino acid changes and undesirable mutations cannot be avoided, but the rate of bystander conversion is lower than the targeted conversion, single-cell sorting of bulk edited cells could potentially isolate clones with only the desired target mutations. Various additional base editor variants have recently been engineered with different deaminases, targeting windows, editing efficiencies and PAM specificities^25,66^. The variants with short activity windows^67,68^ without compromising the editing efficiency may help generate isogenic iPSCs with minimal bystander editing. Adenine base editors with more flexible PAM compatibilities^67^ can be used to address the absence of NGG PAMs at the correct position that limits the number of base conversions. While BEs allow only base transitions, the more recently described prime editor (PE) is versatile and can generate single nucleotide insertions, deletions, transitions and transversions^69^. PE has been used in iPSCs with efficiencies up to 60% ^26,28,67–69^, and single-cell cloning was required to generate cell lines with homozygous mutations^70^. However, BEs have higher base conversion rates and lesser chances of indel formation^71,72^. Additionally, PE requires complex prime editing guide RNA (pegRNA) design. Due to these reasons, PE may be employed when transversion mutations or specific single base pair insertions or deletions are required.

Knocking out the genes that exert pleiotropic functions during differentiation or that function in iPSC self-renewal or survival may obscure analyses of gene function at later stages or render iPSCs unable to propagate. Other concerns while culturing iPSCs with constitutive gene knockout include compensatory effects from other genes, undesired lineage bias, and cellular transformation. As ABE8e expression is Dox regulated in the AAVS1-iABE8e-gRNA system, it can be repeatedly activated for sequential editing of multiple genes to generate tumor models through direct modification of multiple oncogenes. Such a system enables the perturbation of single or multiple genes to investigate their functions in a particular tissue or at a specific time point of differentiation and development. Transient inhibition of p53 has been found to enhance prime editing and cytosine base-editing efficiencies in human pluripotent stem cells^73^. Although the effect of p53 inhibition has not been tested in ABE^73^, tunable ABE expression can avoid constitutive and high-level expression of gene editing enzymes that may activate p53 levels within the cells^33,34^.

In summary, our study demonstrates that the AAVS1-iABE8e iPSC gRNA system is a rapid and effective protocol for generating a bulk population of base-edited iPSCs with homozygous genomic variants. Our results also indicate that integrating Dox-inducible base editor enzymes at the AAVS1 site significantly advances the implementation of base-editing technologies in pluripotent stem cells, making them more accessible for researchers interested in using these cells for developmental biology, disease modeling, drug screening and regenerative medicine. As the bulk population of AAVS1-iABE8e iPSCs with uniform inducible expression of ABE8e could be generated within two weeks without the requirement of single-cell sorting and cloning, such cell lines can be generated with modified ABEs, CBEs and PEs for efficient temporal base editing to create a large number of mutations in iPSCs before or after differentiation to specific cell types.

### Supplementary Information

Supplementary Information.

## Data Availability

NGS data generated in this study have been deposited in the NCBI SRA database under the identifier SRP436643.
