# Supplementary material for: Efficient gene editing in induced pluripotent stem cells enabled by an inducible adenine base editor with tunable expression
Source: Sci Rep. 2023 Dec 11;13:21953. doi: 10.1038/s41598-023-42174-2 (PMC10713686; doi:10.1038/s41598-023-42174-2)
Supplement: Supplementary file 1 — Supplementary Information. [file 41598_2023_42174_MOESM1_ESM.pdf]

Supplemental Figure. S1

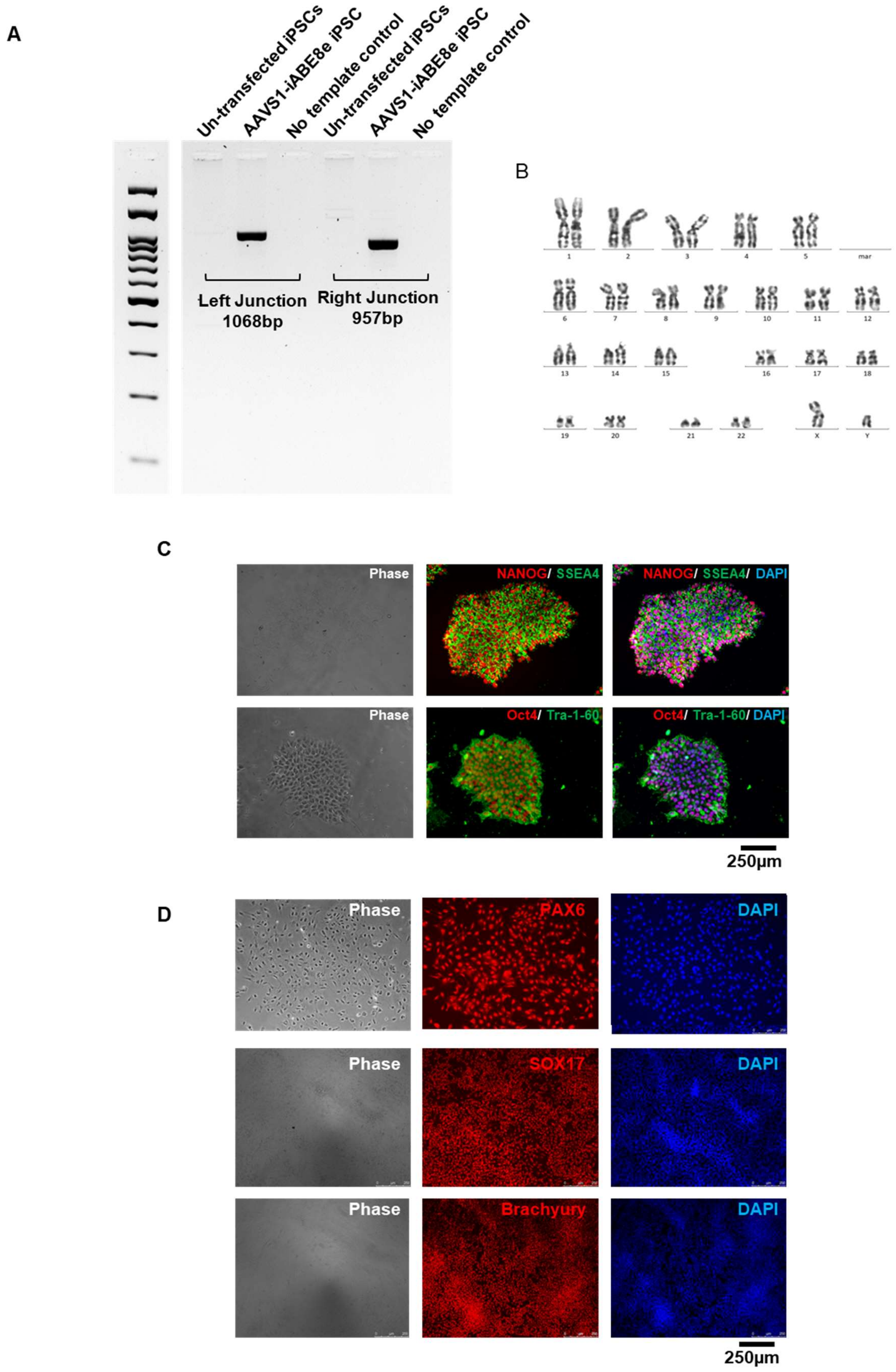

Supplemental Figure. S1. Characterization of AAVS1-iABE8e iPSCs**. (A)** Results of junction PCRs using a forward primer that binds upstream of the DSB site and a reverse primer binding to the exogenous Tet-On-ABE8e cassette (left junction) and a forward primer binding to the Tet-On-ABE8e cassette and reverse primer binding downstream of the DSB site (right junction). **(B)** Karyotyping analysis, revealing no numerical or structural chromosomal abnormalities in the AAVS1-iABE8e iPSCs. **(C)** Immuno-staining of pluripotency markers NANOG. SSEA4, Oct4 and Tra-1-60 in AAVS1-iABE8e iPSCs. Scale bar: 250μm. **(D)** Trilineage differentiation of AAVS1-iABE8e iPSCs confirmed by Immuno-staining of the expression of PAX6 (Ectoderm), SOX17 (Endoderm) and Brachyury (Mesoderm). Nuclei (Blue) were stained with DAPI. Scale bar: 250μm.

Supplemental Figure. S2

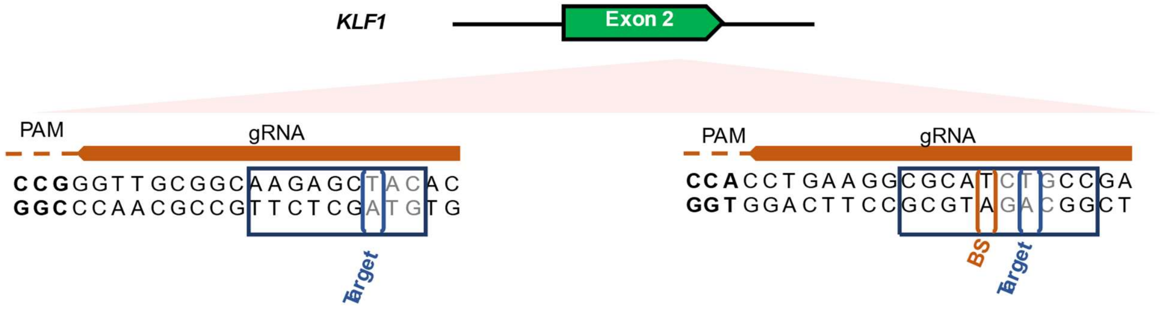

Predicted Targeting Efficiency: Average		
Predicted conversion rates		
Target	Y290H	99.1%

Predicted Targeting Efficiency: Average		
Predicted conversion rates		
Target	L300P	53.1%
BS	L300L	28.7%
Target+BS	L300P	17.3%

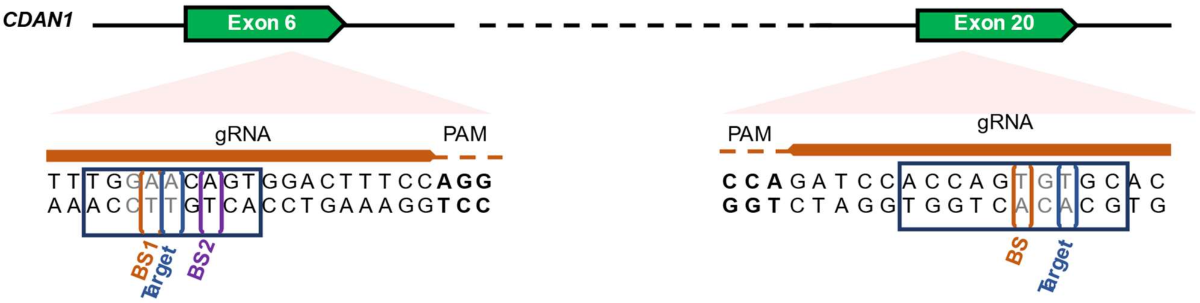

Predicted Targeting Efficiency: Average		
Predicted conversion rates		
Target	F360L	20.0%
BS1	F360S	22.2%
BS2	L359P	22.5%
Target+BS1	F360P	6.6%
Target+BS2	L359P & F360L	4.9%
BS1+BS2	L359P & F360S	5.5%
Target+BS1+BS2	L359P & F360P	1.4%

Predicted Targeting Efficiency: Above Average		
Predicted conversion rates		
Target	T884A	51.9%
BS	T884T	11.9%
Target+BS	T884A	34.6%

Supplemental Figure. S2. Design and analysis of gRNAs used for generating single and multiple mutations in AAVS1-iABE8e iPSCs. gRNAs targeting KLF1 Y290H, KLF1 L300P, CDAN1 F360L and CDAN1 T884A were manually designed and analyzed by BE-Hive for the predicted adenine base conversion efficiencies. The editing windows of the gRNAs are marked by a blue box. The targeted base change is marked 'Target' and the by-stander conversions in the editing window are marked as 'BS1' and 'BS2'. The predicted efficiencies of each conversion and the combinations of conversions are tabulated. The gRNA binding region of the target gene was considered for the total A to G edit quantification.

Supplemental Figure. S3

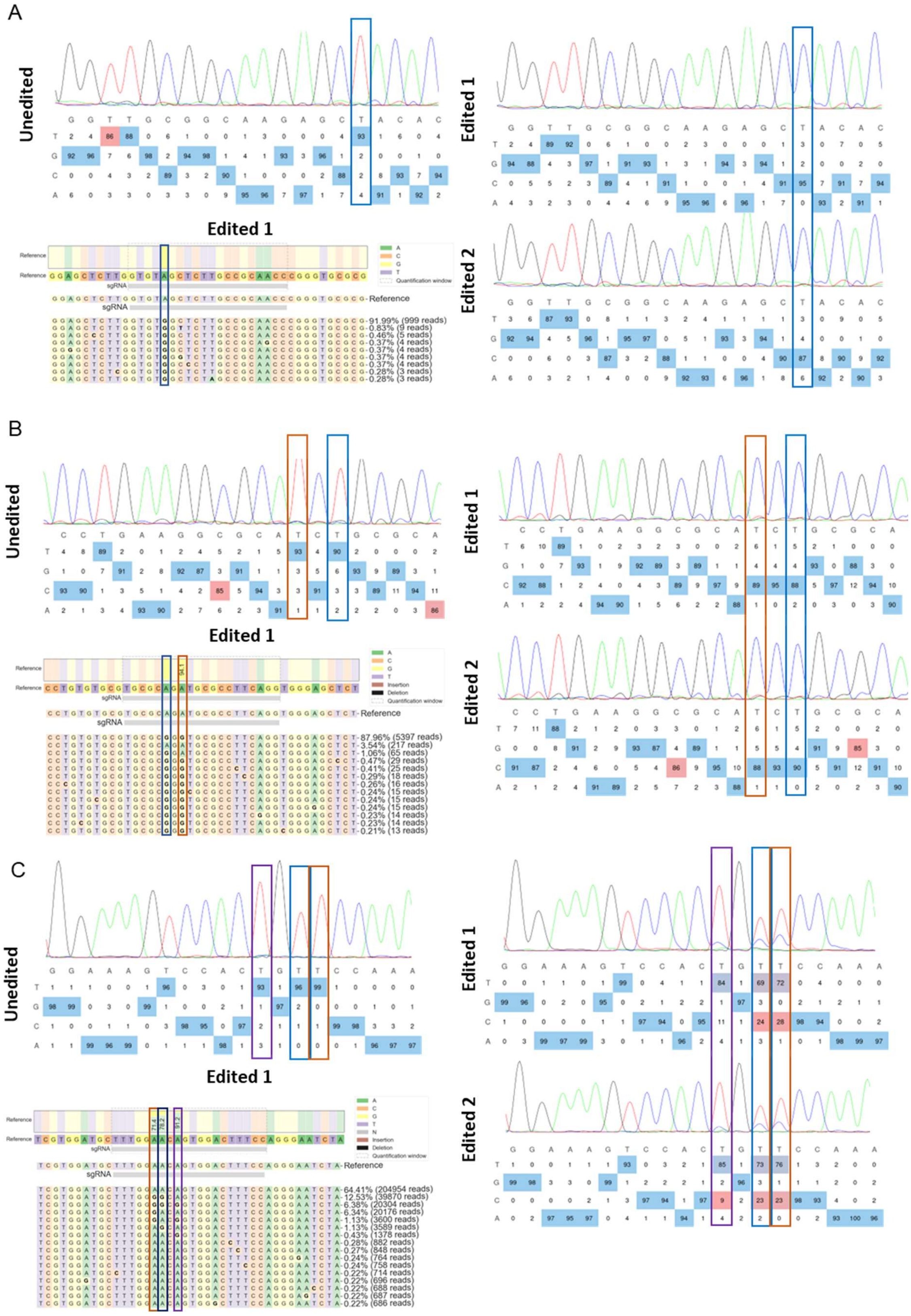

Supplemental Figure. S3. Single locus adenine base editing in AAVS1-iABE8e iPSCs. (A-C) Editing with modified synthetic gRNAs for *KLF1* Y290H **(A)**, *KLF1* L300P **(B)** and *CDAN1* F360L **(C)**. AAVS1-iABE8e iPSCs were electroporated with the three gRNAs separately after pre-treating the cells with 0.5μg/ml doxycycline for 48 hours. Editing was quantified in duplicates (1 and 2) by Sanger sequencing and analysed by EditR. NGS was performed in one of the duplicates and analysed by CRISPResso. The targeted base change is marked 'Target' and any by-stander adenine conversions in the editing window are marked as 'BS'. KLF1 Y290H gRNA had no BS conversion, KLF1 L300P bad one bystander (BS) and CDAN1 F360L had two bystander conversions (BS1 and BS2). The gRNA binding region of the target gene was considered for the total A to G edit quantification**.**

Supplemental Figure. S4

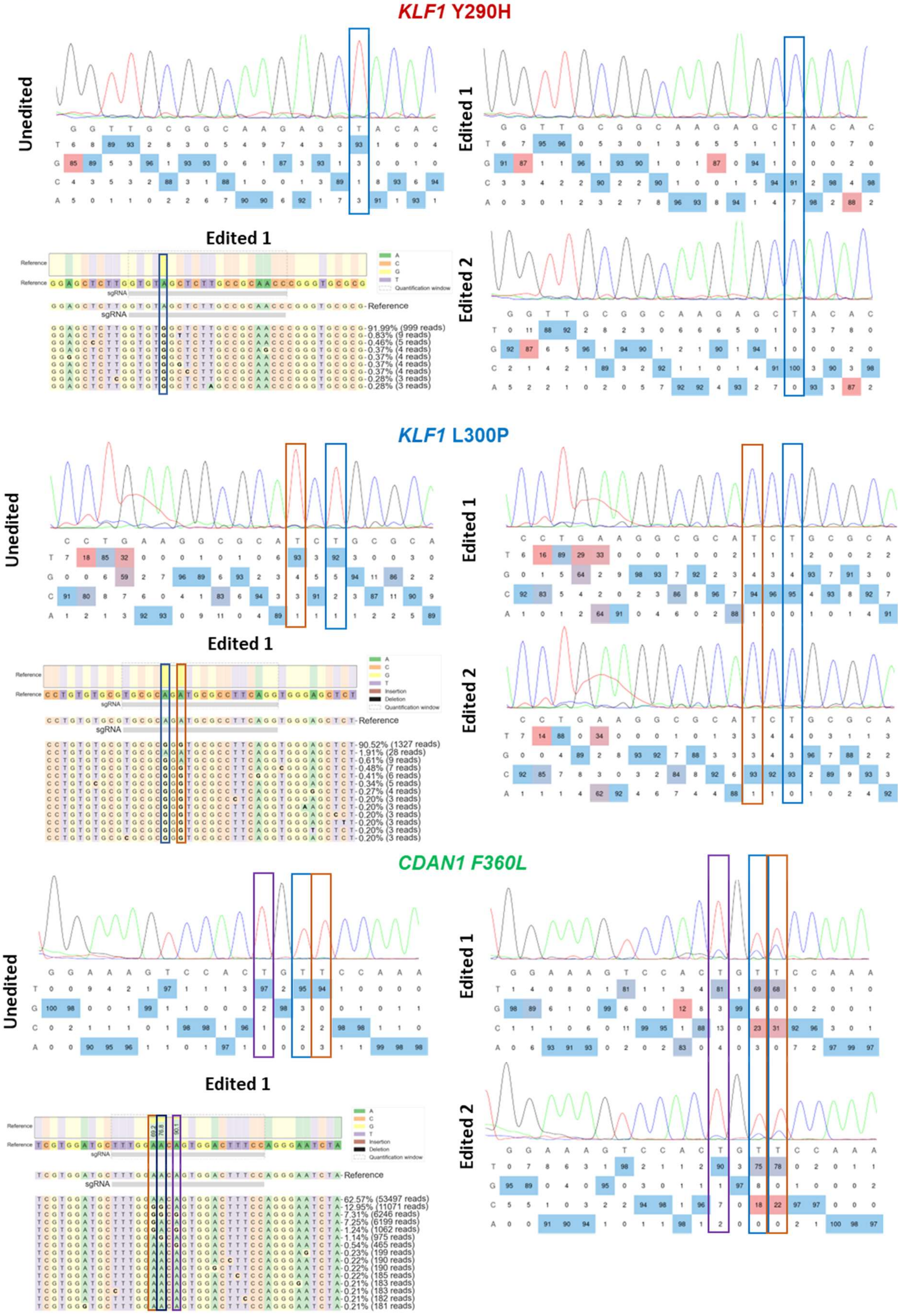

Supplemental Figure. S4. Sequential adenine base editing in AAVS1-iABE8e iPSCs using synthetic sgRNAs. Synthetic modified gRNAs to create *KLF1* Y290H, *KLF1* L300P and *CDAN1* F360L mutations by sequential electroporation of the gRNAs into AAVS1-iABE8e iPSCs. The cells were electroporated with the three gRNAs sequentially after pre-treating the cells with 0.5μg/ml doxycycline for 48 hours. Editing was quantified in duplicates (1 and 2) by Sanger sequencing and analysed by EditR. NGS was performed in one of the duplicates and analysed by CRISPResso. The targeted base change is marked as 'Target' and any by-stander adenine conversions in the editing window are marked as 'BS'. The KLF1 Y290H gRNA did not result in any BS conversion, while KLF1 L300P had one BS conversion and CDAN1 F360L had two BS conversions (BS1 and BS2). The gRNA binding region of the target gene was considered for the total A to G edit quantification.

Supplemental Figure. S5

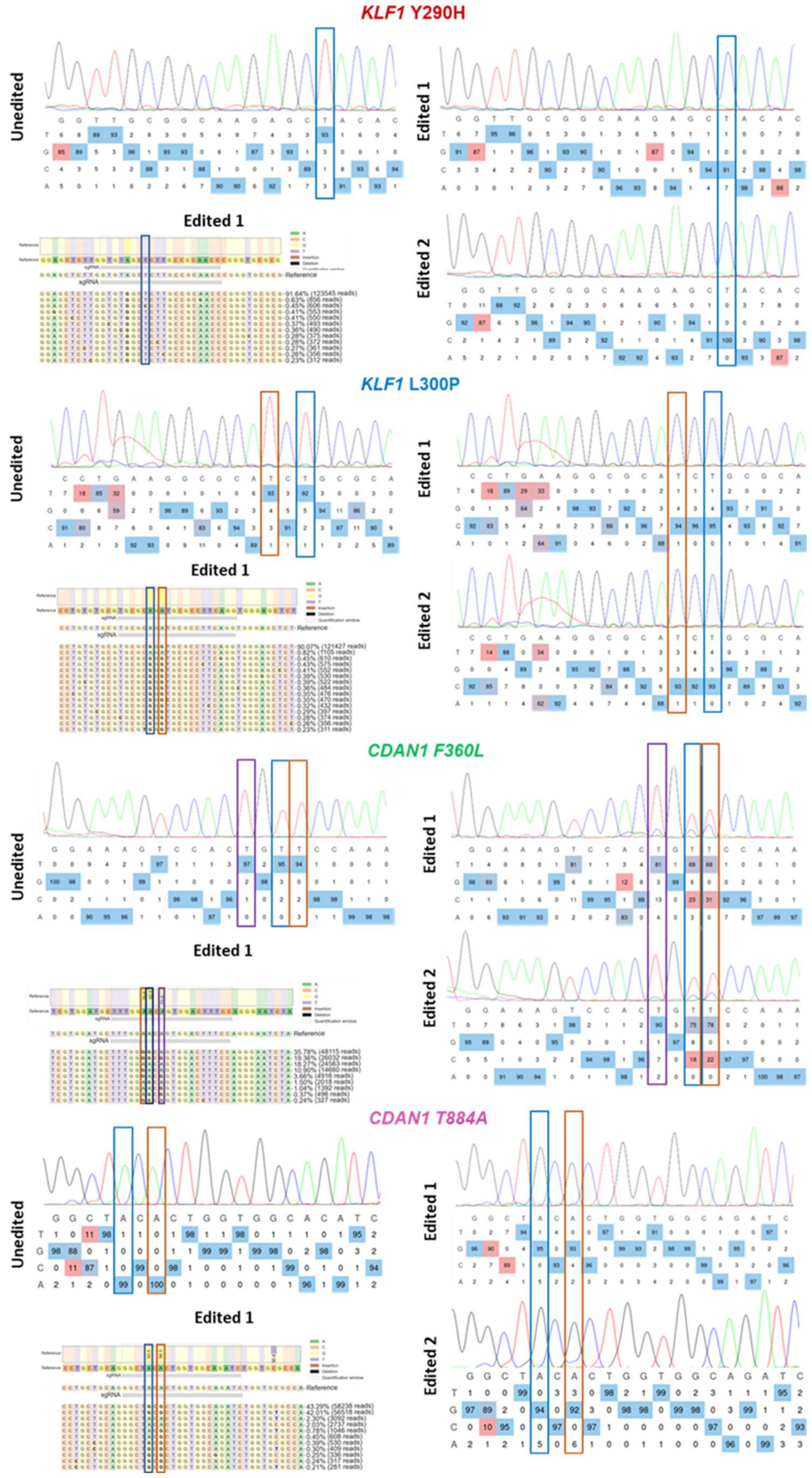

Supplemental Figure. S5. Simultaneous adenine base editing in AAVS1-iABE8e iPSCs using synthetic sgRNAs. Synthetic modified gRNAs to create four mutations, *KLF1* Y290H, *KLF1* L300P, *CDAN1* F360L and *CDAN1* T884A, were electroporated simultaneously into AAVS1-iABE8e iPSCs after pre-treating the cells with 0.5µg/ml doxycycline for 48 hours to induce ABE8e expression. Editing was quantified in duplicates (1 and 2) by Sanger sequencing and analysed by EditR. NGS was performed in one of the duplicates and analysed by CRISPResso. The targeted base change is marked as 'Target', and any by-stander adenine conversions in the editing window are marked as 'BS'. The KLF1 Y290H gRNA did not result in any BS conversion, while KLF1 L300P and CDAN1 T884A had one BS conversion each, and CDAN1 F360L had two BS conversions (BS1 and BS2). The sequence reads within the gRNA binding region were considered for total edit quantification.

Supplemental Figure. S6

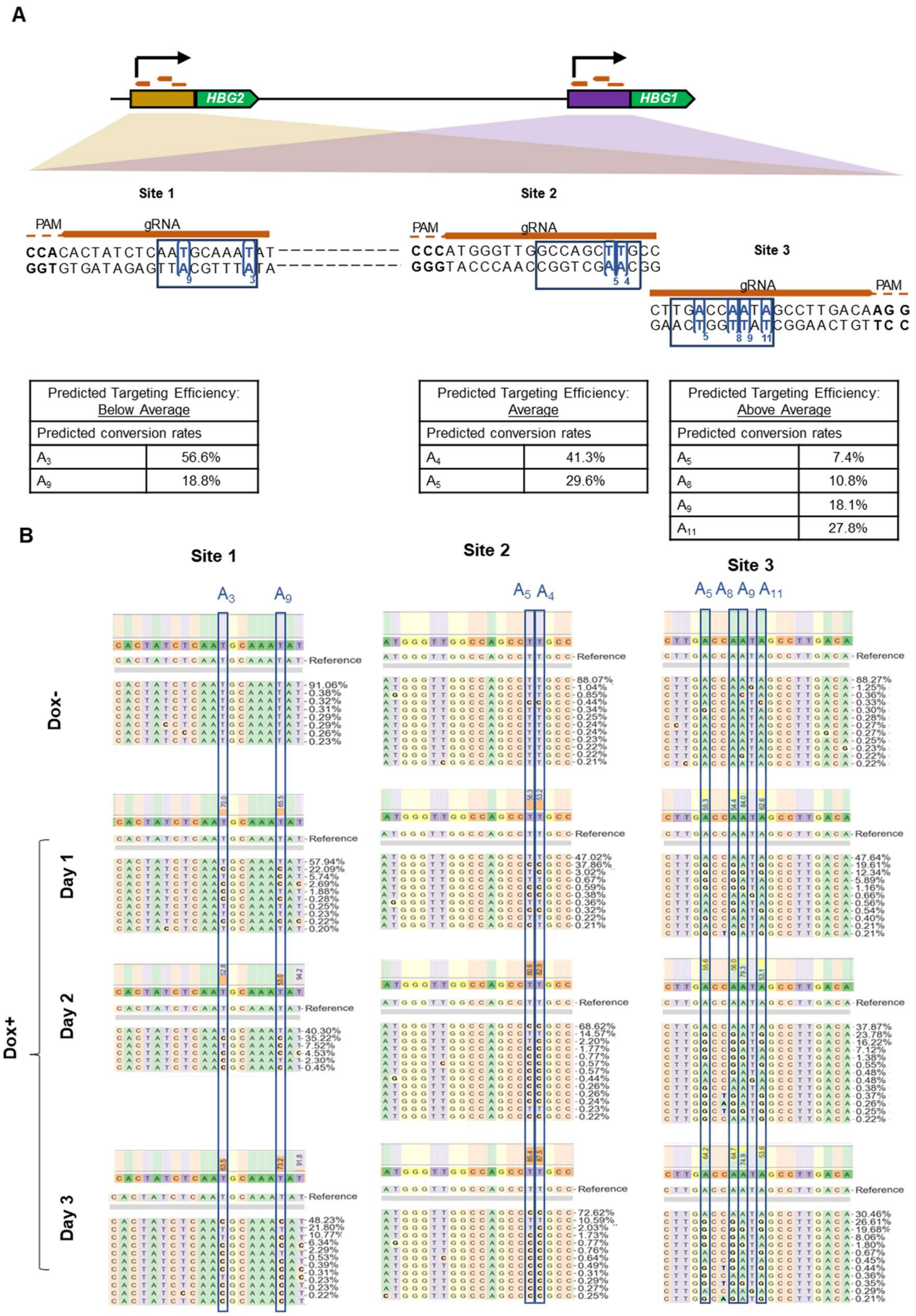

Supplemental Figure. S6. Editing in AAVS1-iABE8e iPSCs with lentivirally expressed gRNAs. **(A)** Schematic representation of gRNAs that target three sites of *HBG1* and *HBG2* promoter regions, which have identical sequences. Each gRNA was designed manually and analysed by Be-Hive to predict their base conversion efficiencies. The predicted efficiencies of each conversion and combinations of conversions are tabulated. The editing windows are marked by dashed boxes, which contain multiple adenines. **(B)** The quantification of editing for three days (day 1, day 2 and day 3) following the induction of ABE8e expression in EGFP+ flow- sorted AAVS1-iABE8e cells transduced with the gRNAs. NGS data analysed by CRISPResso are shown. The adenines in the editing windows that underwent base conversion are marked.

Supplemental Figure. S7

A

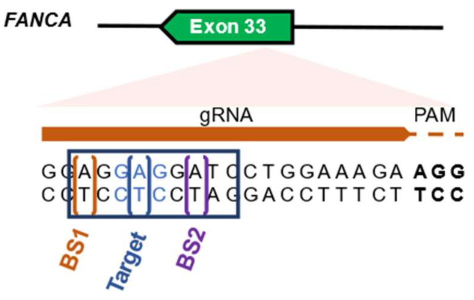

Predicted Overall Targeting Efficiency: Above average		
**Predicted conversion rates**		
Target	L1082P	64.3%
By-stander 1	L1081P	3.6%
By-stander 2	I1083T	10.0%
Target + By-stander 1	L1082P + L1081P	6.0%
Target + Bystander 2	L1082P + I1083T	13.0%
Bystander 1 + Bystander 2	L1081P + I1083T	0.9%
Target + By-stander 1 + By-stander 2	L1082P + L1081P + I1083T	1.1%

B

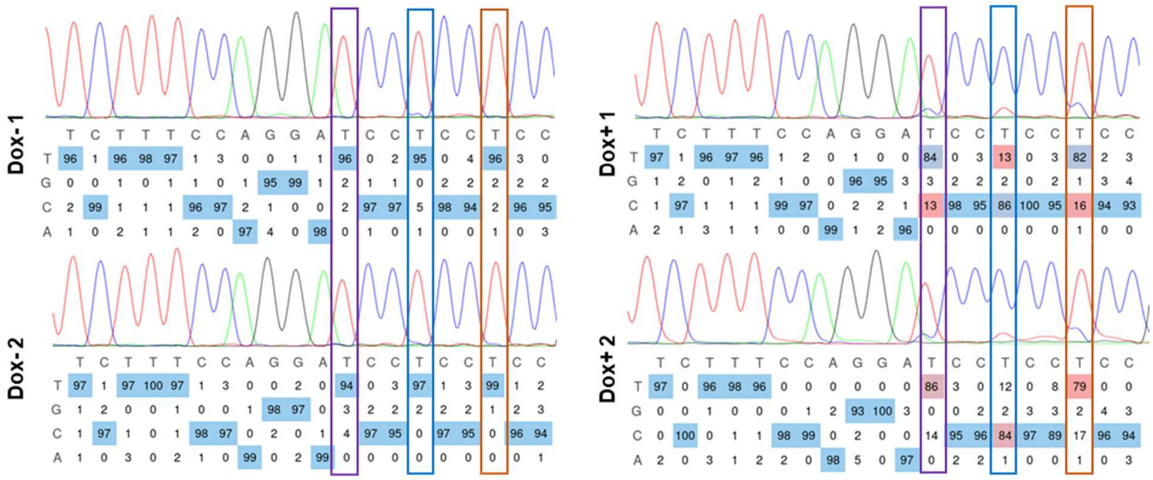

C

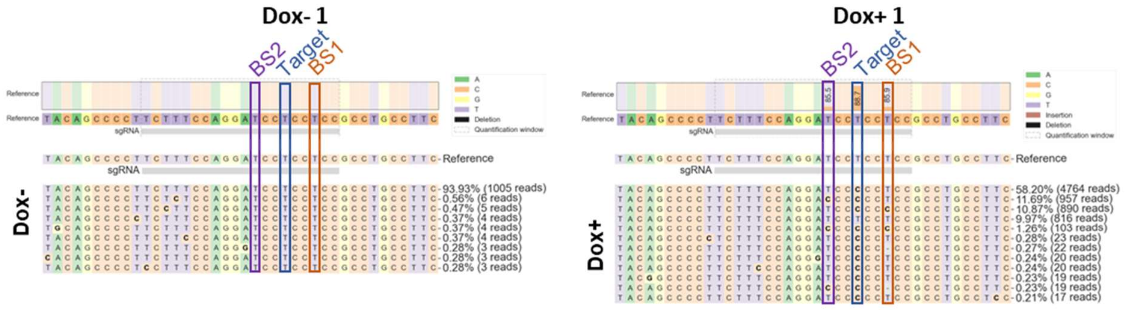

D

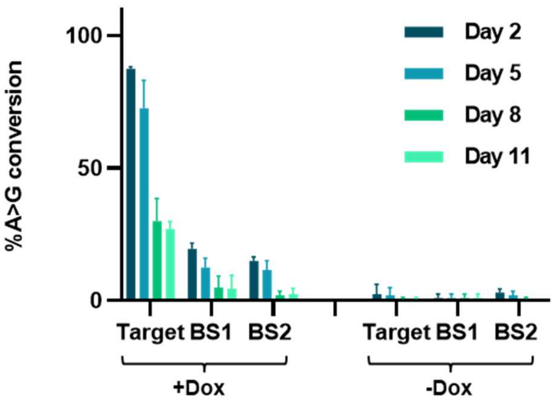

Supplemental Figure. S7. Editing FANCA for isogenic disease modelling of Fanconi anemia (A) gRNA targeting *FANCA* that was manually designed and analysed by Be-Hive for the predicted base conversion efficiencies. The editing window of this gRNA, marked by a blue box, has three adenines. The predicted efficiencies of each conversion and the combinations of conversions is tabulated. **(B)** Sanger sequencing results analyzed by EditR of the targeted region for base editing in the AAVS1-iABE8e iPSCs cultured in the presence and absence of doxycycline and electroporated with the gRNA in duplicates (1 and 2) **(C)** NGS analysis of adenine base conversion in the targeted region for one of the duplicates. The data was analyzed by CRISPResso. The targeted base change is marked 'Target' and the by-stander conversions in the editing window are marked as 'BS1' and 'BS2'. The sequence reads within the gRNA binding region were considered for total edit quantification **(D)** Bar graph showing the decrease in the percentage of mutant cells from the edited population with time as quantified by Sanger sequencing and EditR analysis.

Supplemental Figure. S8

A

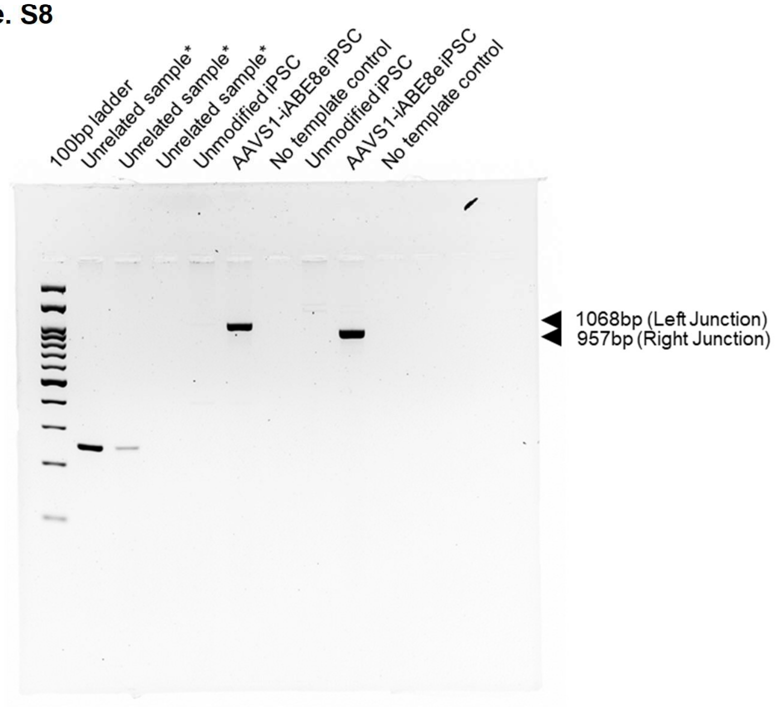

B

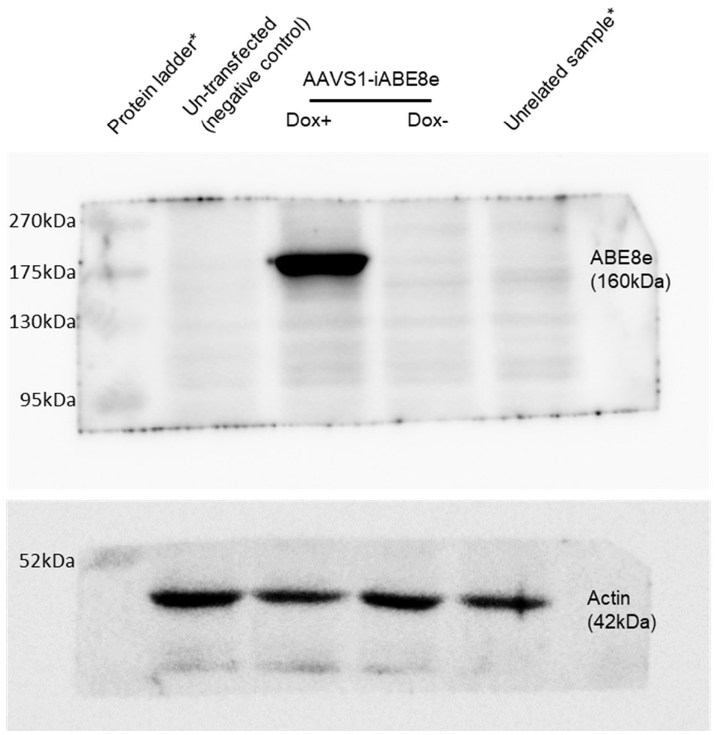

Supplemental Figure. S8. (A) Complete Agarose Gel Electrophoresis for Supplemental Figure S1A. **(B)** Full-length immunoblots for Figure 1C. (*lanes not shown in the main figure).

Supplemental Figure. S9

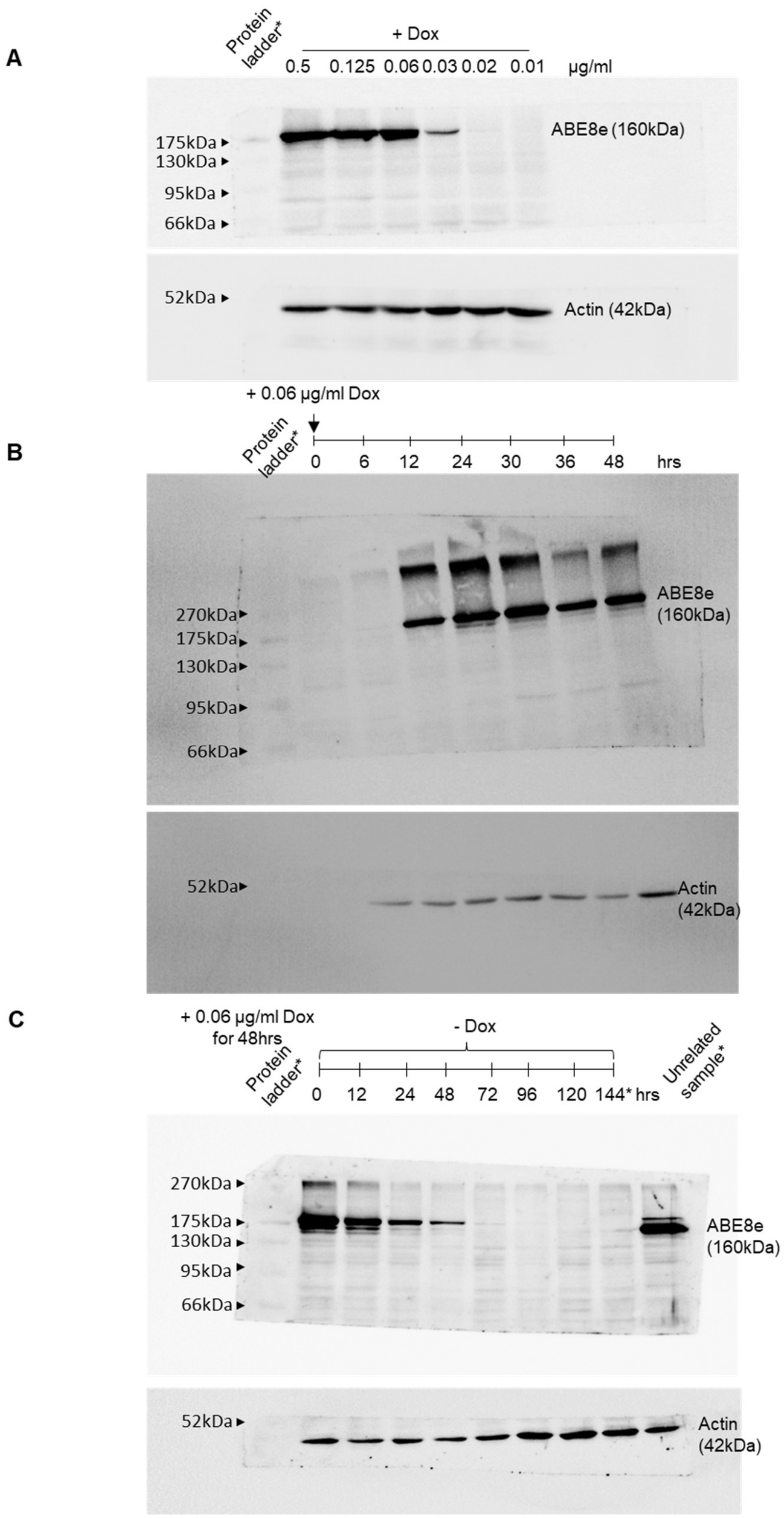

Supplemental Figure. S9. Full-length immunoblots for Figure 2A **(A)**, Figure 2B **(B)** and Figure 2C **(C)** (*lanes not shown in the main figure).

Supplemental Figure. S10

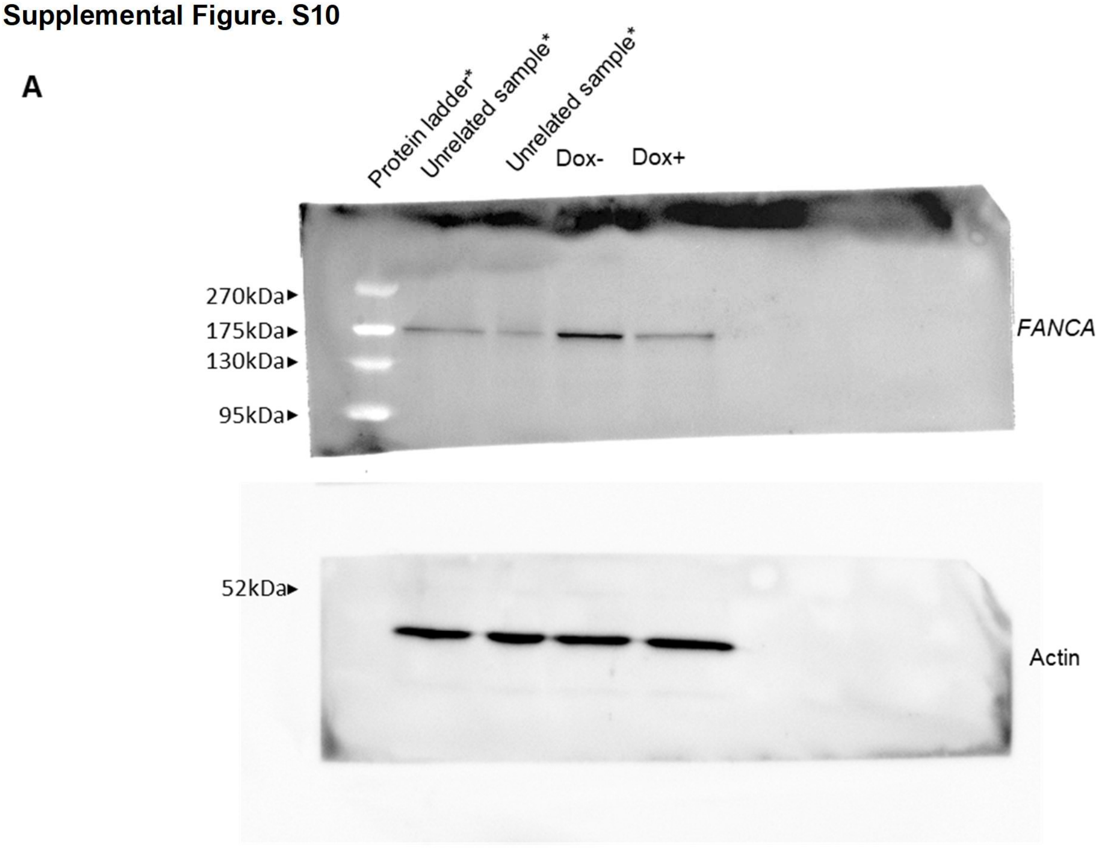

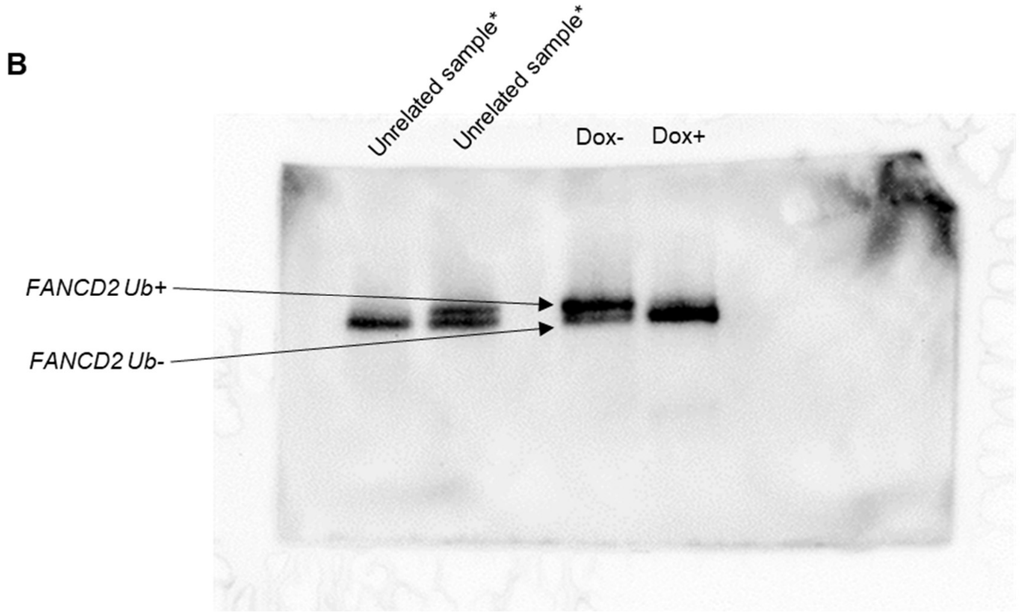

Supplemental Figure. S10. Full-length immunoblots for Figure 6E and 6F. (*lanes not shown in the main figure).

Supplemental Figure. S11

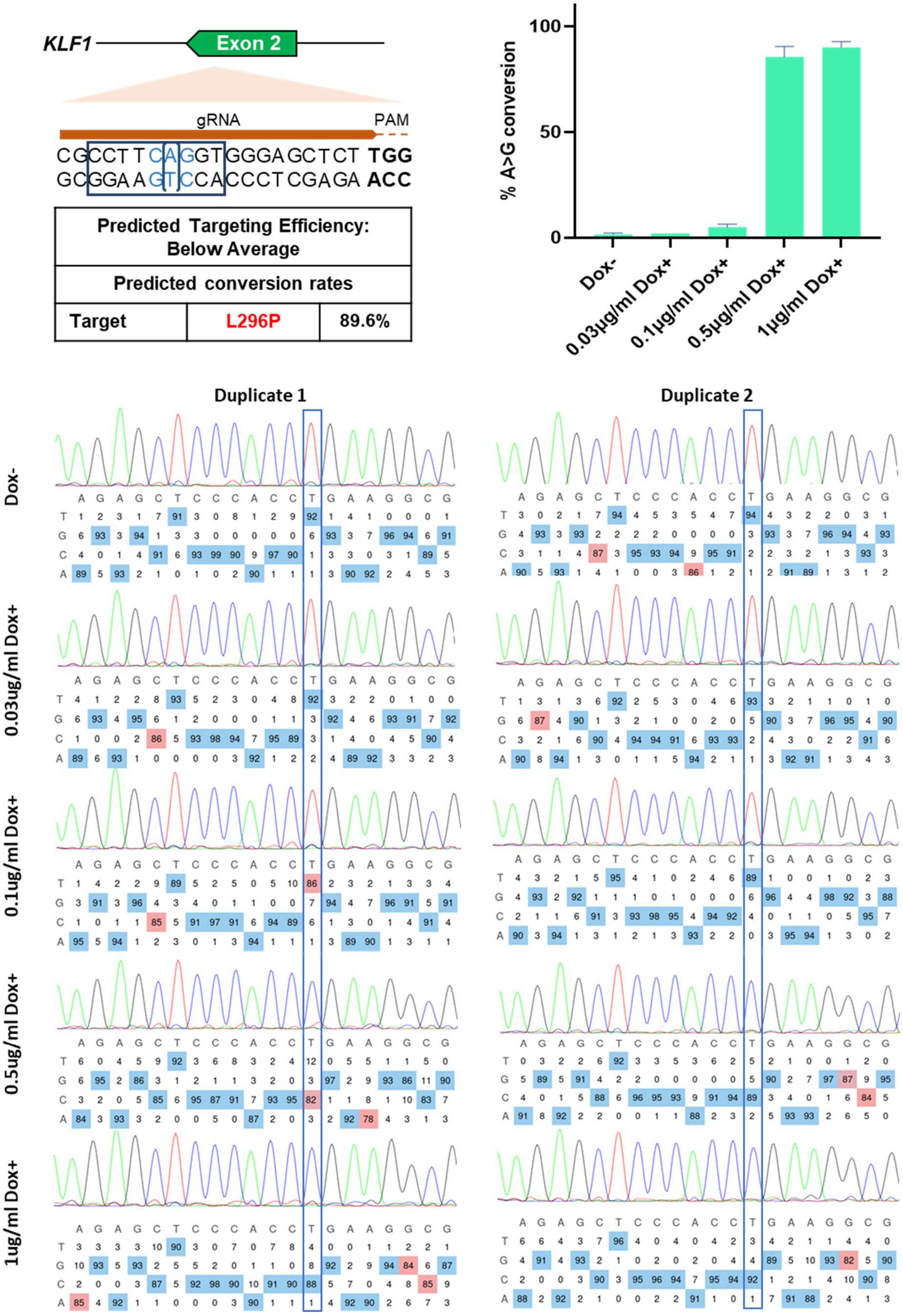

Supplemental Figure. S11. Correlation of doxycycline concentration and editing efficiency (A) gRNA targeting *KLF1* that was manually designed and analysed by Be-Hive for the predicted base conversion efficiencies. The editing window of this gRNA, marked by a blue box, has a single adenine and no by-stander base. The predicted efficiency of conversion is tabulated. **(B)** Sanger sequencing results of the targeted region for base editing in the AAVS1-iABE8e iPSCs cultured in different concentrations of doxycycline and then electroporated with the gRNA (in duplicates). The Sanger sequencing data were analyzed by EditR and the experimental conversion efficiency is shown as a graph (Top left). The data are presented as the mean ± SD of two independent experiments.

Supplemental Figure. S12

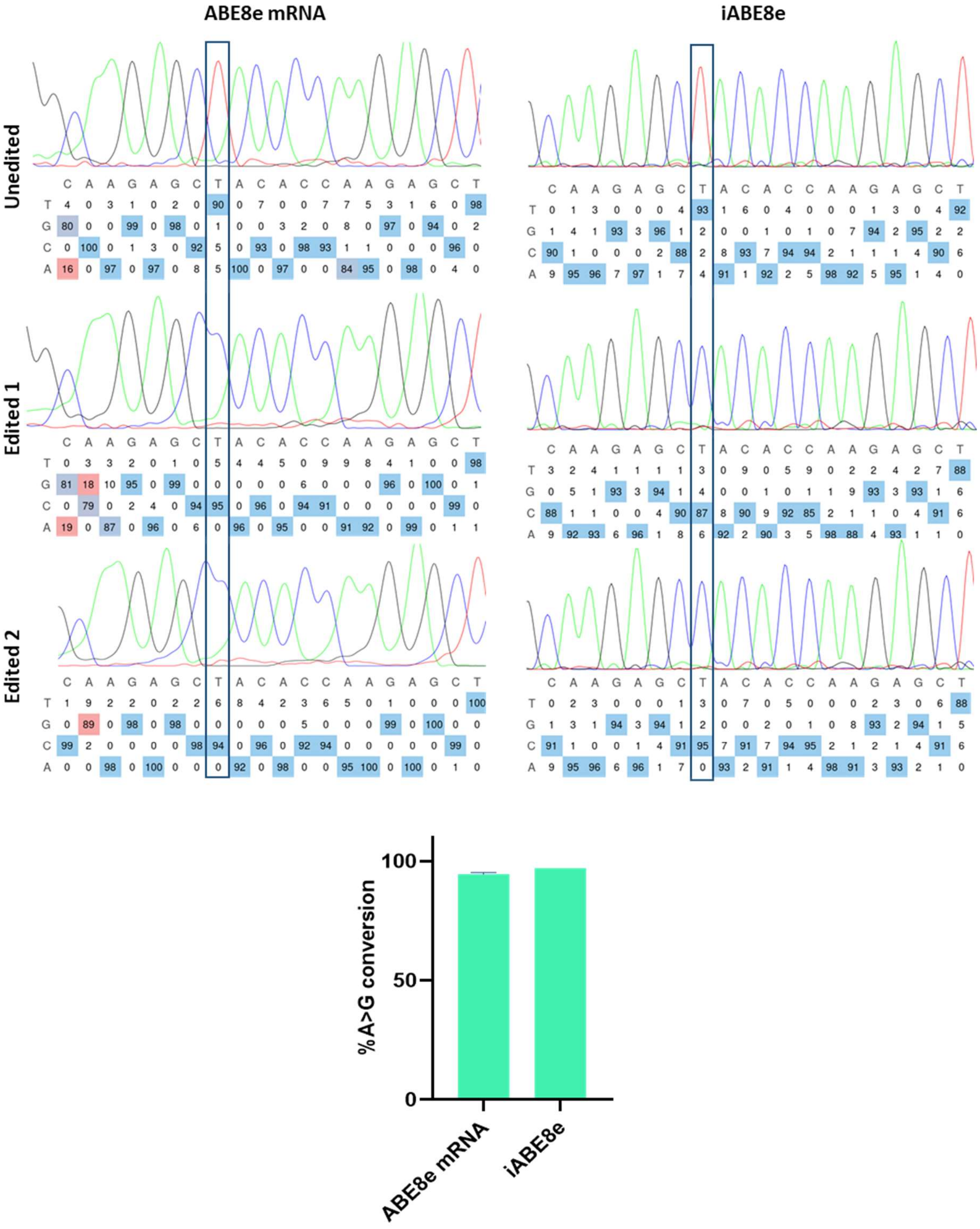

Supplemental Figure. S12. Comparison of editing efficiencies of ABE8e mRNA and iABE8e by the gRNA targeting KLF1 Y290H. Editing quantification was done in duplicates by Sanger Sequencing and EditR analysis. The experimentally determined editing efficiencies are depicted in the bar graph and presented as the mean ± SD of two independent experiments.

Supplemental Figure. S13

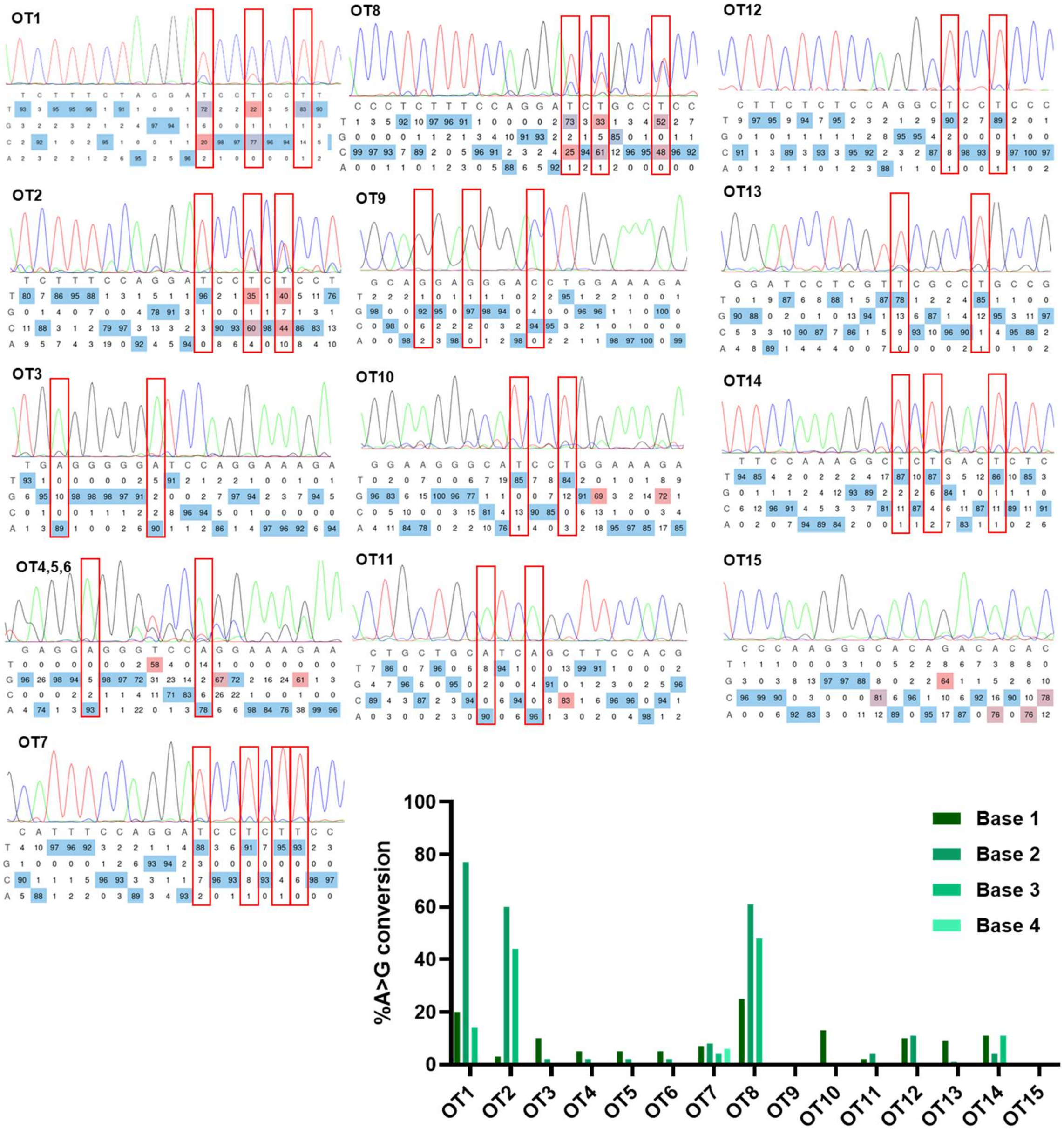

Supplemental Figure. S13. Off-target analysis for the gRNA against FANCA L1082P. Top 10 off-targets (OT1-OT10) along with 5 protein coding regions (OT11 to OT15) were amplified and sequenced, Sanger Sequencing data are analyzed by EditR and the base conversion efficiency is shown as a bar graph.

Supplemental Figure. S14

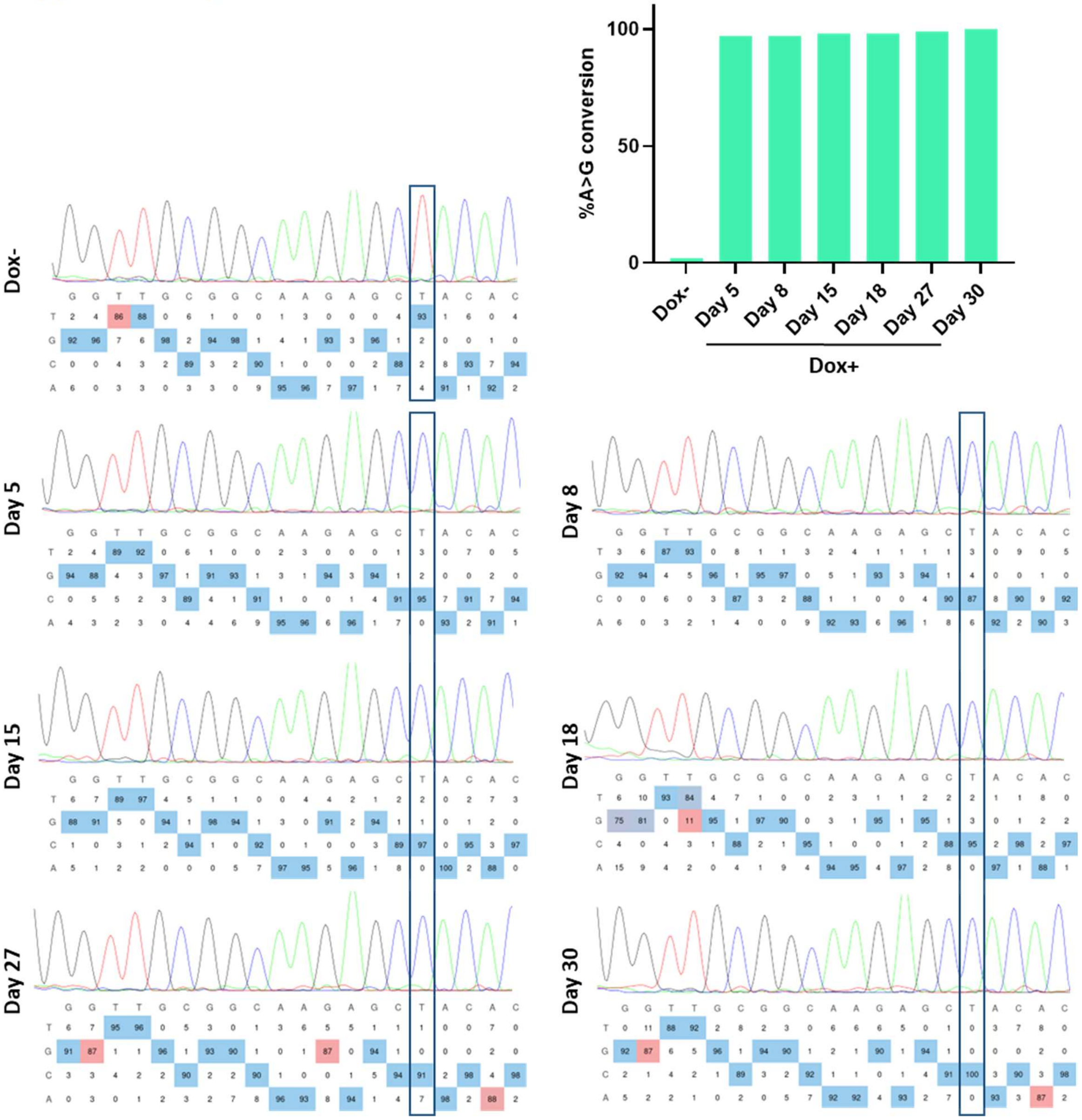

Supplemental Figure. S14. Estimation of the persistence of mutation over a prolonged iPSC culture. Base edited iPSC pool containing predominantly homozygous KLF1 Y290H mutant cells was cultured for 30 days and Sanger sequencing was performed on Day 5, 8, 15, 18, 27 and 30 after electroporation. Data was analysed by EditR.

Table S1: List of oligos used in this study

Oligo name	Sequence (5'-3')	Purpose
**Gibson Assembly Primers**		
iABE8e fragment 1 F	CTCGTAAACTTAAGGTTAATATGAAACGGACAGCCGACGG	To generate the fragment 1 of ABE8e for cloning in pAAVS1-TRE-Cas9- puro-polyA-CAG-rtTA plasmid
iABE8e fragment 1 R	AGTAGTCCTCTTTCAGCTGCTTCACGGTCAC	
iABE8e fragment 2 F	GCAGCTGAAAGAGGACTACTTCAAGAAAATCGAG	To generate the fragment 2 of ABE8e for cloning in pAAVS1-TRE-Cas9- puro-polyA-CAG-rtTA plasmid
iABE8e fragment 2 R	GGCGATCGATTGCGGCCGCATCAATGGTGATGGTGATGATG	
**Junction PCR primers**		
iABE8e Junction PCR Left F	CTGCCGTCTCTCTCCTGAGT	To detect of integration of iABE8e casette
iABE8e Junction PCR Left R	GTGGGCTTGTACTCGGTCAT	
iABE8e Junction PCR Right F	TGTGGGGTGGAGATATCAGC	
iABE8e Junction PCR Right R	GGGGTCCAGGCCAAGTAG	
**Modified gRNAs for electroporation**		
CDAN1 T884A sgRNA	CUUGACCAAUAGCCUUGACA	gRNA to target CDAN1 T884A chr15:42,728,806T>C
CDAN1 T884A sgRNA	CUUGACCAAUAGCCUUGACA	gRNA to target CDAN1 T884A chr15:42,728,806T>C
CDAN1 F360L sgRNA	UUUGGAACAGUGGACUUUCC	gRNA to target CDAN1 F360L chr15:42,735,158A>G
KLF1 Y290H sgRNA	GUGUAGCUCUUGCCGCAACC	gRNA to target KLF1 Y290H chr19:12,885,362A>G
KLF1 L296P sgRNA	AGAGCUCCCACCUGAAGGCG	gRNA to target KLF1 Y290H chr19:12,885,343A>G
KLF1 L300P sgRNA	UGCGCAGAUGCGCCUUCAGG	gRNA to target KLF1 L300P chr19:12,885,331A>G
FANCA L1082P sgRNA	GGAGGAGGAUCCUGGAAAGA	gRNA to target FANCA L1082P chr16:89,748,762A>G
**Lentiviral gRNAs#**		
HBGp Site 1 gRNA oligo F	**CACCG**CTTGACCAATAGCCTTGACA	To clone gRNA against HBG1 and HBG2 promoter Site 1 into pLKO.5.sgrNA.EFS.GFP lentiviral vector
HBGp Site 1 gRNA oligo R	**AAAC**TGTCAAGGCTATTGGTCAAG**C**	
HBGp Site 2 gRNA oligo F	**CACCG**ATATTTGCATTGAGATAGTG	To clone gRNA against HBG1 and HBG2 promoter Site 2 into pLKO.5.sgrNA.EFS.GFP lentiviral vector
HBGp Site 2 gRNA oligo R	**AAAC**CACTATCTCAATGCAAATAT**C**	
HBGp Site 3 gRNA oligo F	**CACCG**GGCAAGGCTGGCCAACCCAT	To clone gRNA against HBG1 and HBG2 promoter Site 2 into pLKO.5.sgrNA.EFS.GFP lentiviral vector
HBGp Site 3 gRNA oligo R	**AAAC**ATGGGTTGGCCAGCCTTGCC**C**	
**Sanger Sequencing/ NGS primary PCR primers***		
CDAN1 T884A NGS F	**TACACGACGCTCTTCCGATCT**TTTTCAGCCTCTTTCCGATT	To detect CDAN1 T884A mutation by Sanger sequencing and NGS
CDAN1 T884A NGS R	**AGACGTGTGCTCTTCCGATCT**GCTGGGAACACAAGATCTCC	
CDAN1 F360L NGS F	**TACACGACGCTCTTCCGATCT**TCAGTGAGCAAGCCTCCTTT	To detect CDAN1 F360L mutation by Sanger sequencing and NGS
CDAN1 F360L NGS R	**AGACGTGTGCTCTTCCGATCT**ATGAGAATGAGGCCCAAATG	
KLF1 Y290H, L296P and L300P NGS F	**TACACGACGCTCTTCCGATCT**CAGGTGTGATAGCCGAGACC	To detect KLF1 Y290H and KLF1 L300P mutation by Sanger sequencing and NGS
KLF1 Y290H, L296P and L300P NGS R	**AGACGTGTGCTCTTCCGATCT**CAACCCTTCTTCCCCTGTAA	
FANCA L1082P NGS F	**TACACGACGCTCTTCCGATCT**CTGAGAGTCTGAGCCCTTGG	To detect FANCA L1082P mutation by Sanger sequencing and NGS
FANCA L1082P NGS R	**AGACGTGTGCTCTTCCGATCT**GTGGAAGAACTGCTCGCATC	
FANCA L1082P OT1 F	**TACACGACGCTCTTCCGATCT**ACTCTCCATGACTCTGCACC	To detect FANCA L1082P off-target base conversions by Sanger sequencing and NGS
FANCA L1082P OT1 R	**AGACGTGTGCTCTTCCGATCT**GGTAGACAGAGAGGGTTCCG	
FANCA L1082P OT2 F	**TACACGACGCTCTTCCGATCT**CCAGAGAGTGTGGGCAAC	
FANCA L1082P OT2 R	**AGACGTGTGCTCTTCCGATCT**CCCTTAAAAGCTCTTCATTGCC	
FANCA L1082P OT3 F	**TACACGACGCTCTTCCGATCT**AAAGGAGGAAGGAGAGCTGG	
FANCA L1082P OT3 R	**AGACGTGTGCTCTTCCGATCT**CCCCACTCCACTTTATTTTCCT	
FANCA L1082P OT4, 5,6 F	**TACACGACGCTCTTCCGATCT**AGCATGGGATCCGAGAAACA	
FANCA L1082P OT4, 5,6 R	**AGACGTGTGCTCTTCCGATCT**GGCCTTCCAGATCCTTTAACAG	
FANCA L1082P OT7 F	**TACACGACGCTCTTCCGATCT**TGCACAAAGGTCCGAGAGT	
FANCA L1082P OT7 R	**AGACGTGTGCTCTTCCGATCT**CACTGCCTCTCCTCAGCTTC	
FANCA L1082P OT8 F	**TACACGACGCTCTTCCGATCT**CTTGGAGAACGGAGGGAAGT	
FANCA L1082P OT8 R	**AGACGTGTGCTCTTCCGATCT**TGAGGCTGCTGAAGATACACA	
FANCA L1082P OT9 F	**TACACGACGCTCTTCCGATCT**GCTTCCTCCGCATTCATGG	
FANCA L1082P OT9 R	**AGACGTGTGCTCTTCCGATCT**GTGACCGGTTGTTTCTGCAT	
FANCA L1082P OT10 F	**TACACGACGCTCTTCCGATCT**GCTTAGAACAATCCTGACACACA	
FANCA L1082P OT10 R	**AGACGTGTGCTCTTCCGATCT**CAGACTGCATGAATGACACTGT	
FANCA L1082P OT11 F	**TACACGACGCTCTTCCGATCT**CGTGGCTAACTCAACTTCCA	
FANCA L1082P OT11 R	**AGACGTGTGCTCTTCCGATCT**GAAGAGTTGTTGCAGCAGGG	
FANCA L1082P OT12 F	**TACACGACGCTCTTCCGATCT**TGTGTGTGGGAGCTGAATCT	
FANCA L1082P OT12 R	**AGACGTGTGCTCTTCCGATCT**TGCCCCACCGAACTCTTC	
FANCA L1082P OT13 F	**TACACGACGCTCTTCCGATCT**GCTGTCTATCGAAACATTGCCA	
FANCA L1082P OT13 R	**AGACGTGTGCTCTTCCGATCT**TTGCTGAGCTGTGGTTGAAG	
FANCA L1082P OT14 F	**TACACGACGCTCTTCCGATCT**GGGCTACAGATGTGCATGAC	
FANCA L1082P OT14 R	**AGACGTGTGCTCTTCCGATCT**TCCTTCCTGCCTGAAATTGC	
FANCA L1082P OT15 F	**TACACGACGCTCTTCCGATCT**GATCAGGGAGCCAAAGGTGA	
FANCA L1082P OT15 R	**AGACGTGTGCTCTTCCGATCT**GGGGCTGATGATGGTCCTTA	
HBGp Site 1, 2, and 3 Seq F	**TACACGACGCTCTTCCGATCT**ACAAAAGAAGTCCTGGTATC	To detect mutaions in the HBG1 and HBG2 promoter sites by Sanger sequencing
HBGp Site 1, 2 and 3 Seq R	**AGACGTGTGCTCTTCCGATCT**CTTCCCAGGGTTTCTCCTCC	
HBGp Site 1, 2, and 3 NGS F	**TACACGACGCTCTTCCGATCT**GGAATGACTGAATCGGAACAA	To dectect mutaions in the HBG1 and HBG2 promoter sites by NGS
HBGp Site 1, 2 and 3 NGS R	**AGACGTGTGCTCTTCCGATCT**GACGTTCCAGAAGCGAGTGT	
**NGS Secondary PCR primers$**		
NGS secondary primer F	**AATGATACGGCGACCACCGAGATCTACAC*NNNNNNNN***ACACTCTTTCCC**TACACGACGCTCTTCCGATCT**	Secondary PCR primers for NGS
NGS secondary primer R	**CAAGCAGAAGACGGCATACGAGAT*NNNNNNNN***GTGACTGGAGTTC**AGACGTGTGCTCTTCCGATCT**	
**RT-qPCR primers**		
ACTIN qPCR F	GACGACATGGAGAAAATCTG	To detect expression of β-Actin
ACTIN qPCR R	ATGATCTGGGTCATCTTCTC	
Cas9 qPCR F	AAAGACCGAGGTGCAGACAG	To detect expression of ABE8e
Cas9 qPCR R	ACCAGCACAGAATAGGCCAC	

Note:
\# **CACCG** in the forward oligo and **AAAC** in the 5' end and **C** in the 3' end of the reverse oligo are overhangs for cloning into pLKO5.sgRNA.EFS.GFP vector.
* **TACACGACGCTCTTCCGATCT** and **AGACGTGTGCTCTTCCGATCT** are tag sequences in the forward and reverse primers, respectively, for the primary PCR for NGS.
$ **AATGATACGGCGACCACCGAGATCTACAC** and **CAAGCAGAAGACGGCATACGAGA** are tag sequences in the forward and reverse primers, respectively, used for the secondary PCR for NGS. ***NNNNNNNN*** represent different index sequences in the forward and reverse primers for for multiplexing.

Table S2: List of off-targets of FANCA L1082P sgRNA and their conversion rates

Serial No.	Off-target Sequence	Mismatch Count	Locus Description	CFD score	Conversion of bases in the editing window (%)				Amino acid change
					Base 1	Base 2	Base 3	Base 4	
OT1	GAAGGAGGATCCTAGAAAGA**TGG**	2	intron:RP11-753H16.3	0.63	20.0	77.0	14.0		-
OT2	AGGAGAGGATCCTGGAAAGA**TGG**	3	intergenic:RP11-307L14.2-Y_RNA	0.49	3.0	60.0	44.0		-
OT3	TGAGGGGGATCCAGGAAAGA**GGG**	3	intergenic:FARSA-CALR	0.49	10.0	2.0			-
OT4	GAGGAGGGTCCAGGAAAGAA**GGG**	2	intron:ADAMTS7P2	0.59	5.0	2.0			-
OT5	GGAGGAGGGTCCAGGAAAGA**AGG**	2	intron:ADAMTS7P1	0.46	5.0	2.0			-
OT6	GGAGGAGGGTCCAGGAAAGA**AGG**	2	intergenic:RP11-114H24.3-ADAMTS7P3	0.46	5.0	2.0			-
OT7	GGAAGAGGATCCTGGAAATG**CGG**	3	intergenic:SLC14A2/RP11-116O18.3-SLC14A2	0.46	7.0	8.0	4.0	6.0	-
OT8	GGAGGCAGATCCTGGAAAGA**GGG**	2	intron:RP6-65G23.1	0.45	25.0	61.0	48.0		-
OT9	GCAGGAGGGACCTGGAAAGA**GGG**	3	intergenic:RP11-35O7.1-CTD-2029E14.1	0.40	0.0	0.0	0.0		-
OT10	GGAAGGGCATCCTGGAAAGA**TGG**	3	intron:RP11-321F6.1	0.40	13.0	0.0			-
OT11	CGTGGAAGCTGATGCAGCAG**TGG**	3	exon:PROSER1	0.50	6.0	1.0	6.0		S568P: 6% (Benign*)<br>S566P: 6% (Benign*)
OT12	GGGAGGAGCCTGGAGAGAAG**GGG**	3	exon:THRA	0.48	10.0	11.0			- (3' UTR)
OT13	CGGCAGGCGAACGAGGATCC**GGG**	3	exon:MRPS25/NR2C2	0.42	5.0	9.0	1.0		L547P: 5% (Pathogenic*)<br>V545A: 9% (Undetermined significance*)
OT14	GAGAGTCAGAGCCTTTGGAA**AGG**	3	exon:RNF144A	0.42	11.0	4.0	11.0		- (3' UTR)
OT15	GTGTGTCTGTGCCCTTGGGA**GGG**	3	exon:NOP2	0.41	0.0				No A in editing window

*predicted by Varsome
